# CD10^−^/ALDH^−^ cells are the sole cisplatin-resistant component of a novel ovarian cancer stem cell hierarchy

**DOI:** 10.1038/cddis.2017.379

**Published:** 2017-10-19

**Authors:** Brendan Ffrench, Claudia Gasch, Karsten Hokamp, Cathy Spillane, Gordon Blackshields, Thamir Mahmoud Mahgoub, Mark Bates, Louise Kehoe, Aoibhinn Mooney, Ronan Doyle, Brendan Doyle, Dearbhaile O'Donnell, Noreen Gleeson, Bryan T Hennessy, Britta Stordal, Ciaran O'Riain, Helen Lambkin, Sharon O'Toole, John J O'Leary, Michael F Gallagher

**Affiliations:** 1 Department of Histopathology, Trinity College Dublin, Central Pathology Laboratory, St. James’s Hospital, Dublin 8, Ireland; 2 Pathology Research Laboratory, Coombe Women and Infant’s University Hospital, Dublin 8, Ireland; 3 Smurfit Institute of Genetics, Trinity College Dublin, Dublin 2, Ireland; 4 Department of Obstetrics and Gynaecology, Trinity College Dublin, Trinity Centre for Health Sciences, St. James’s Hospital, Dublin 8, Ireland; 5 School of Biological Sciences, Dublin Institute of Technology, Kevin Street, Dublin, Ireland; 6 Department of Clinical Medicine, Trinity College Dublin, St. James’s Hospital, Dublin 8, Ireland; 7 Department of Molecular Medicine, Royal College of Surgeons in Ireland, Dublin 2, Ireland; 8 Department of Natural Sciences, Middlesex University, Hendon, London, UK

## Abstract

It is long established that tumour-initiating cancer stem cells (CSCs) possess chemoresistant properties. However, little is known of the mechanisms involved, particularly with respect to the organisation of CSCs as stem-progenitor-differentiated cell hierarchies. Here we aimed to elucidate the relationship between CSC hierarchies and chemoresistance in an ovarian cancer model. Using a single cell-based approach to CSC discovery and validation, we report a novel, four-component CSC hierarchy based around the markers cluster of differentiation 10 (CD10) and aldehyde dehydrogenase (ALDH). In a change to our understanding of CSC biology, resistance to chemotherapy drug cisplatin was found to be the sole property of CD10^−^/ALDH^−^ CSCs, while all four CSC types were sensitive to chemotherapy drug paclitaxel. Cisplatin treatment quickly altered the hierarchy, resulting in a three-component hierarchy dominated by the cisplatin-resistant CD10^−^/ALDH^−^ CSC. This organisation was found to be hard-wired in a long-term cisplatin-adapted model, where again CD10^−^/ALDH^−^ CSCs were the sole cisplatin-resistant component, and all CSC types remained paclitaxel-sensitive. Molecular analysis indicated that cisplatin resistance is associated with inherent- and adaptive-specific drug efflux and DNA-damage repair mechanisms. Clinically, low CD10 expression was consistent with a specific set of ovarian cancer patient samples. Collectively, these data advance our understanding of the relationship between CSC hierarchies and chemoresistance, which was shown to be CSC- and drug-type specific, and facilitated by specific and synergistic inherent and adaptive mechanisms. Furthermore, our data indicate that primary stage targeting of CD10^−^/ALDH^−^ CSCs in specific ovarian cancer patients in future may facilitate targeting of recurrent disease, before it ever develops.

Tumour-initiating cells within the heterogeneous tumour are referred to as ‘cancer stem cells’ (CSCs) owing to their stem cell-like properties of self-renewal, differentiation and (malignant) tissue genesis.^1, 2, 3^ Many studies have shown that CSCs from different malignancies are chemoresistant.^4^ CSCs are potential clinical targets, particularly for the treatment of refractory disease, but CSC targeting has not been efficiently translated to the clinic.^1, 5^ This may be due to the complex organisation of CSCs as stem-progenitor-differentiated cell hierarchies. Current models indicate that, *in vivo*, a highly plastic, immature CSC sits at the apex of a unidirectional tree structure. The apex CSC produces more mature, less plastic CSCs, which are referred to as (committed) ‘progenitors’, which produce mature, specialised (‘differentiated’) cells, which form the bulk of the tumour.^2^ Contemporary CSC theory indicates that the developing tumour is a collection of hierarchically arranged apex CSCs, progenitor CSCs and differentiated cells. However, the degree to which different CSC types contribute to chemoresistance is unknown.

Platinum-based chemotherapy, a cornerstone of cancer treatment, targets DNA by generating intrastrand and interstrand crosslinks (ICLs), triggering apoptosis at the G_1_/S phase cell cycle checkpoint.^6^ Cisplatin resistance is associated with two mechanisms. First, multi-drug resistance (MDR) genes, which have a strong association with chemoresistant cells, facilitate active efflux of chemotherapy drugs out of the cell. The best characterised of these is the ATP-binding cassette (ABC) transporter family.^7^ Second, DNA-damage response (DDR) mechanisms facilitate efficient repair of ICLs via the Fanconi anaemia (FA) pathway.^8, 9, 10^ Briefly, the FA core complex (FANCA/B/C/E/F/G/L/M) recognises and binds the ICL and recruits and activates the key FANCD2–FANCI heterodimer, which facilitates unhooking of the ICL. Subsequently, BRCA2 (breast cancer, early onset 2)-driven homologous recombination, sometimes referred to as the FA/BRCA pathway, regenerates targeted DNA, facilitating continuation through the cell cycle and a resumption of proliferation. Importantly, FA/BRCA pathway DDR is regulated by several ubiquitination/de-ubiquitination steps, which form part of the ubiquitin proteasome system (UPS).^11^ In contrast, taxane chemotherapeutics target microtubule stability, which leads to apoptosis at G_2_/M of the cell cycle. As cells do not have a microtubule repair system, taxane resistance is associated with mechanisms such as tubulin isotype mutations that affect taxane–microtubule binding.^12^ It is likely that identification and targeting of specific chemoresistance mechanisms in specific CSC types could enhance treatment of refractory disease.

In this study, single-cell analysis facilitated generation and validation of a novel four-component CSC hierarchy from a cisplatin-sensitive, treatment-naive ovarian cancer model based around the expression of the stem cell markers cluster of differentiation 10 (CD10) and aldehyde dehydrogenase (ALDH). Despite the dogma that all CSCs are broadly chemoresistant, all four CSC subtypes were found to be paclitaxel sensitive, while cisplatin resistance was the property of only CD10^−^/ALDH^−^ CSCs. The CSC hierarchy adapted to short- and long-term cisplatin treatment, which was associated with specific expression of MDR and DDR mechanisms. Together, these data indicate, for the first time, that CSC chemoresistance is not universal and can be CSC-type and drug-type specific, which suggests important, novel considerations for clinical targeting strategies.

## Results

### CD10^+^/ALDH^+^ CSCs are the apex population in a new, four-component, cisplatin-sensitive, treatment-naive ovarian CSC network

**Figure 1:**
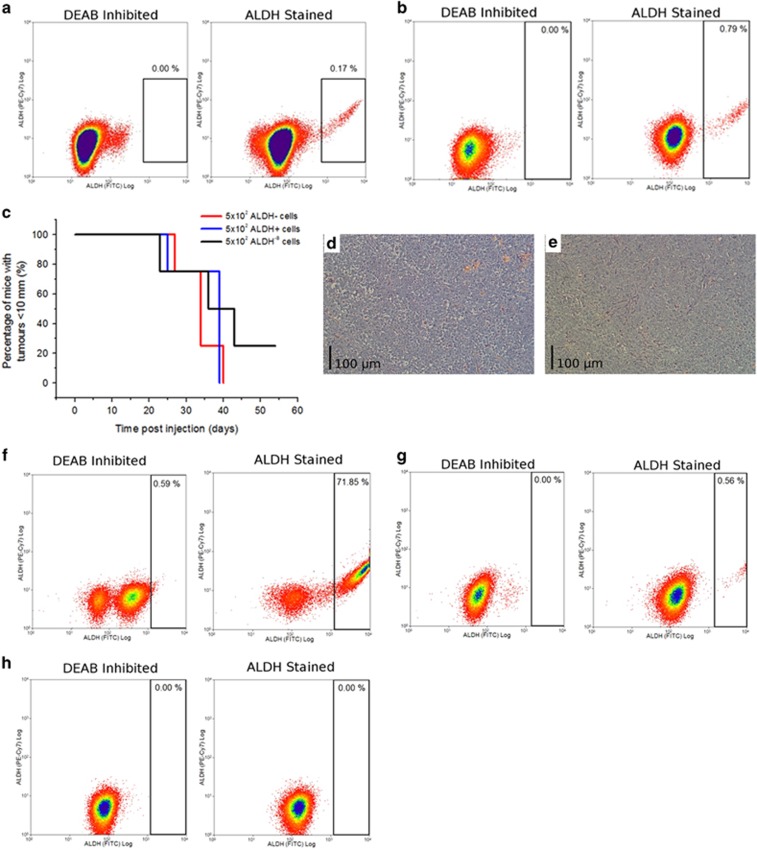
Identification of a novel ALDH ovCSC. The cisplatin-sensitive, treatment-naive cancer model A2780 and its cisplatin-adapted counterpart A2780cis were screened for the presence of putative CSC populations based on the expression of CD44, CD113, CD117 and CXCR4, as well as Hoechst efflux capacity (Side Population assay) and ALDH activity (Supplementary Data 1). Both cell types contained a population with strong ALDH activity (**a**): A2780, (**b**): A2780cis, flow cytometry using ALDH inhibitor DEAB as a negative control. ALDH^+^ and ALDH^−^ cells were isolated from the A2780 model by FACS and found to efficiently generate xenograft tumours *in vivo* (**c**), *n*=4 for each cell type that were confirmed as representative of ovarian cancer by pathological analysis (H+E staining, (**d**): ALDH^+^, (**e**): ALDH^−^). Cells of each type were plated singly, allowed to develop into colonies and then assessed for the presence of ALDH^+^ and ALDH^−^ cells (SCAD assay). (**f**) SCAD assays demonstrated that ALDH^+^ cells could produce ALDH^+^ and ALDH^−^ cell types, validating them as CSCs, *n*=4. (**g**) Some ALDH^−^ clones (termed ALDH^−A^, *n*=4) were found to generate both cell types, validating them as CSCs also. (**h**) However, other ALDH^−^ clones (termed ALDH^−B^, *n*=6) were found to produce only ALDH^−^ cells. When isolated by FACS, ALDH^−B^ cells were also found to efficiently generate xenograft tumours *in vivo* (**c**). Similar results were generated for the A2780cis model (Supplementary Data 1).These data indicated the presence of a complex CSC network, the elucidation of which required identification of a second marker, a process that is described in Supplementary Data 2. All experiments were conducted in three biological replicates and statistical significance was determined as described in Materials and Methods section

**Figure 2:**
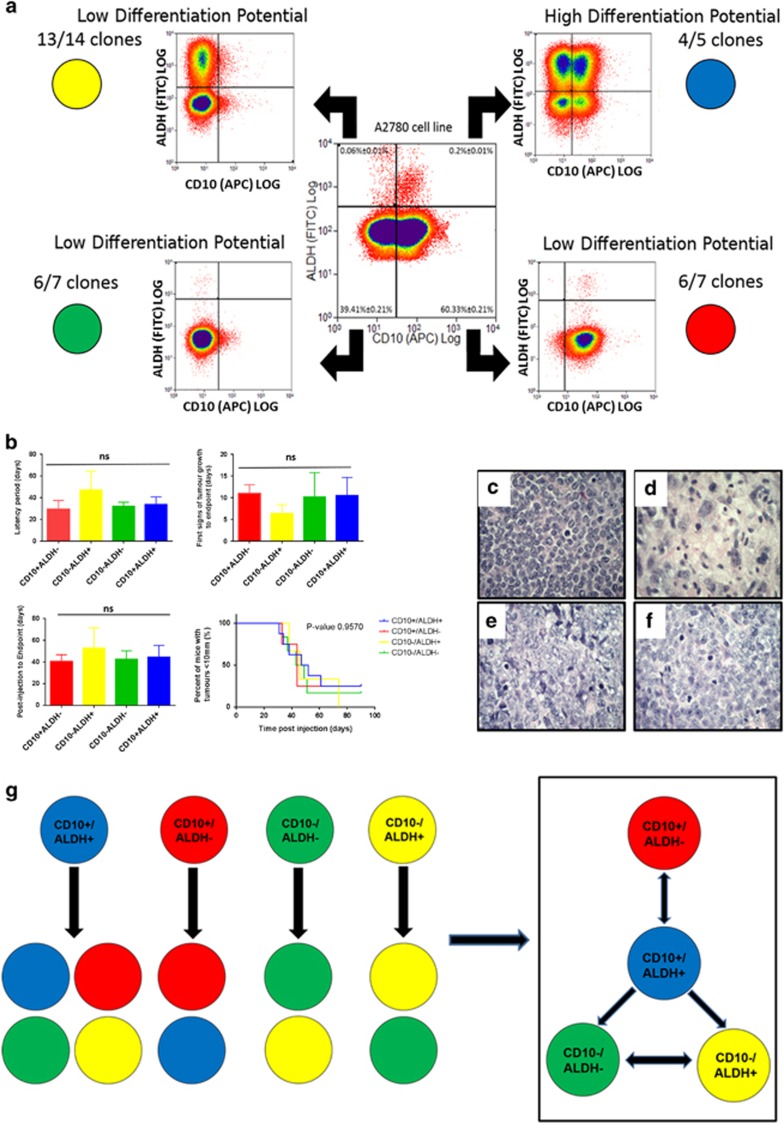
CD10^+^/ALDH^+^ is the apex component of a novel ovCSC network. CD10 was identified as an ALDH^−A^ marker as described in Supplementary Data 2, resulting in identification of four putative CSC populations via flow cytometry (CD10^+^/ALDH^+^, CD10^−^/ALDH^−^, CD10^+^/ALDH^−^, CD10^−^/ALDH^+^: **a**, centre). Each of the four populations was similarly capable (no significant (NS) difference in latency period, first sight of tumour growth, time from injection to end point or percentage of mice generating tumours) of generating xenograft tumours from as little as 90 cells, validating them as true CSCs (**b**; Population mean comparison using analysis of variance test. Data shown are mean±S.D. of *n*⩾3 mice). Histological analysis demonstrated an ovarian cancer-like pathology (**c**: CD10^−^/ALDH^−^, **d**: CD10^+^/ALDH^−^, **e**: CD10^−^/ALDH^+^, **f**: CD10^+^/ALDH^+^), with no consistent differences between the populations. All four populations were plated as single cells and assessed for their relationship to one another via SCAD assay (**a**). (**g**) This analysis was summarised and modelled and identified CD10^+^/ALDH^+^ as the most potent, apex CSC. Unusually, this apex CSC could be produced by less potent CD10^−^/ALDH^+^ CSCs. The two additional low-potency CSCs were capable of producing one another. Rather than the standard hierarchical, tree structure, this novel CD10/ALDH model is apparently a CSC Network

The study began with the identification of the CD10 and ALDH markers through a screening and validation process (described in detail in Supplementary Data 1) in both the cisplatin-sensitive treatment-naive cancer model A2780 (Figure 1) and its long-term cisplatin-adapted counterpart A2780cis. A2780/A2780cis is a highly characterised cisplatin-sensitive/-adapted model that was originally derived from a treatment-naive ovarian cancer patient of unknown histology/pathology. Four populations were identified within the A2780 cell line: CD10^+^/ALDH^−^ (60.33±0.21%), CD10^−^/ALDH^−^ (39.41±0.21%), CD10^+^/ALDH^+^ (0.2±0.01%), and CD10^−^/ALDH^+^ (0.06±0.01%) (Figure 2a, Supplementary Data 1). Each population was isolated by FACS, validated as a true CSC via generation of a xenograft tumour from <100 cells (Figures 2b–f), and assessed for their relationships to one another via single-cell asymmetric division (SCAD: described in detail in Supplementary Data 1) assay (Figure 2a). This data indicated the presence of a novel, four-component ovarian CSC (ovCSC) network, with a small CD10^+^/ALDH^+^ population as the most potent, apex CSCs (Figure 2g). This hierarchy is not a common tree structure and is non-linear: progenitor CSCs (CD10^−^/ALDH^−^ and CD10^−^/ALDH^+^) can produce one another, and one, CD10^+^/ALDH^−^, can produce the apex CSC. It is further noted that all four populations showed at least one clone that did not display asymmetric division in the SCAD assay (Figure 2a), suggesting the presence of additional rare, non-CSC populations.

### CD10^−^/ALDH^−^ CSCs are the only cisplatin-resistant component of the CD10/ALDH hierarchy

**Figure 3:**
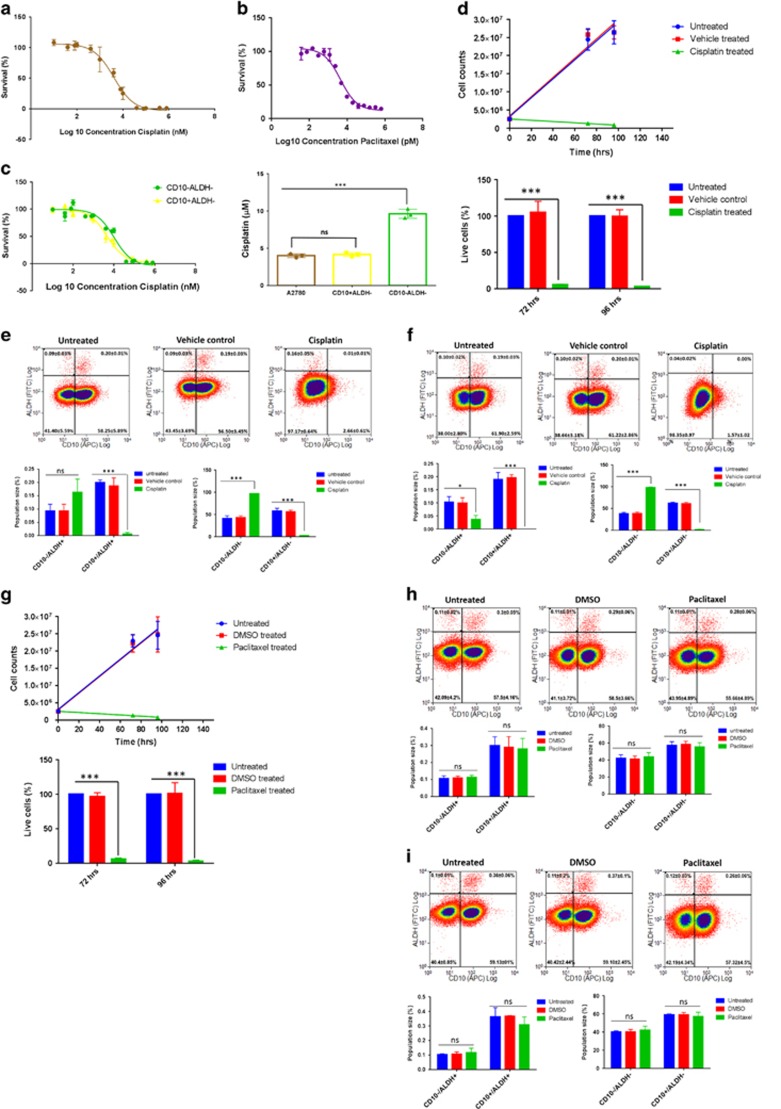
CD10^−^/ALDH^−^ CSCs are the chemoresistant component of the CD10/ALDH ovCSC network. Dose–response analysis was used to calculate IC_50_s of (**a**) 4.1±0.2 *μ*M for cisplatin, *n*=3; and (**b**) 4.6±1.0 nM for paclitaxel, *n*=3 for A2780 cells. To elucidate the relationship between the CD10/ALDH network and chemoresistance, A2780 cells were treated with cisplatin for (**d** and **e**) 72 and (**f**) 96 h, and the effect on the four populations was then assessed via flow cytometry (**e** and **f**). Although untreated and DMSO vehicle-treated control cells were unaffected (**d**–**f**), cisplatin treatment resulted in a substantial cell death (**d**). Flow cytometry indicated that this was due to substantial cell death in the CD10^+^/ALDH^+^, CD10^−^/ALDH^+^ and CD10^+^/ALDH^−^ populations (**e** and **f**). In contrast, a proportional increase in the size of the CD10^−^/ALDH^−^ population was observed (**e** and **f**). This was confirmed by statistical analysis (bar charts, unpaired Student’s *t*-test, mean population size (%)±S.D. of *n*=3, NS=not significant, ***P-value<0.0001). Notably, by 96 h (**d** and **f**) the apex CD10^+^/ALDH^+^ CSC has been depleted to the point of being undetectable. Although the CD10^+^/ALDH^+^ and CD10^−^/ALDH^+^ populations were too small to facilitate toxicology assays, cisplatin dose–response curves (**c**: IC50, left panel; statistical analysis, right panel) showed that the CD10^−^/ALDH^−^ CSCs (IC_50_=9.6±0.6 *μ*M; *n*=3) were proportionally cisplatin resistant and the CD10^+^/ALDH^−^ CSCs proportionally cisplatin sensitive (IC_50_=4.2±0.3 *μ*M; *n*=3). In contrast, A2780 cells were found to have a much lower tolerance for paclitaxel (**b**: IC_50_=4.6±1.0 nM). (**h**) Seventy-two and (**i**) 96 h 4.6 nM paclitaxel treatment was found to result in substantial cell death (**g**), which affected all four populations uniformly. Together, these data indicate that CD10^−^/ALDH^−^ CSCs are the sole cisplatin-resistant component of this ovCSC network. As these cells had not been exposed to cisplatin previously, this is deemed to be inherent chemoresistance. However, these data indicate that the entire ovCSC network is highly sensitive to paclitaxel

The CSC network was next assessed for cisplatin sensitivity. A2780 cells were exposed to their 48 h cisplatin IC_50_ (Figure 3a) for 72 or 96 h, and the effect on each hierarchy component was assessed by flow cytometry. At 72 h, only the CD10^−^/ALDH^−^ population displayed cisplatin resistance, while the other three subpopulations showed substantial cell death relative to untreated and vehicle-treated controls (Figures 3d–f). By 96 h the CD10^+^/ALDH^+^ apex CSC population was undetectable, while the CD10^−^/ALDH^−^ population increased from 38.66±3.18% (treatment naive) to 98.35±0.97% post cisplatin-treatment (Figures 3e and f). Although the CD10^+^/ALDH^+^ and CD10^−^/ALDH^+^ populations were too small to facilitate toxicology analysis, it was subsequently shown that the CD10^−^/ALDH^−^ population had a cisplatin IC_50_ of 9.6±0.6 *μ*M compared with only 4.1±0.2 *μ*M for the A2780 parent cell line (Figure 3c). Finally, an identical experiment was performed for paclitaxel treatment (Figures 3b and g–i). Although there was substantial cell death during the assay (Figure 3g), the flow cytometric profile for the network was unaffected (Figures 3h and i), indicating that all populations were equally paclitaxel sensitive.

### CD10^−^/ALDH^−^ CSCs are the dominant population of a three-component cisplatin-adapted ovCSC hierarchy

**Figure 4:**
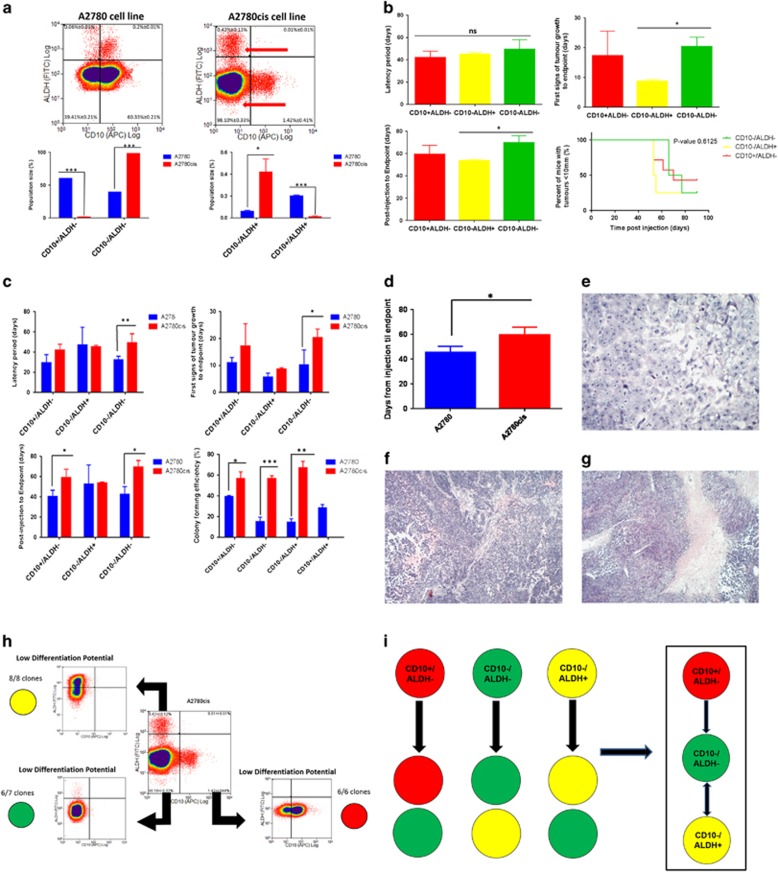
Cisplatin adaptation hard-wires changes to the CD10/ALDH ovCSC network. (**a**) Flow cytometric analysis indicated that the A2780cis model contained only three CD10/ALDH populations, as CD10^+^/ALDH^+^ cells were undetectable. This suggests that the altered flow cytometric profile observed when the A2780 model was treated with cisplatin for 96 h has been hard-wired in the A2780cis model. (**b**) All three populations could generate xenograft tumours from as little as 90 cells, validating them as true CSCs. Some differences were observed between CD10^−^/ALDH^−^ and CD10^−^/ALDH^+^ CSCs (first sight of tumour growth, time from injection to end point, **b**, *P-value<0.05). There was no significant (NS) difference in latency period. (**c**) Additionally, it was noted that tumour formation and colony formation was slower in A2780cis CSCs compared with A2780 CSCs. A2780cis populations were found to more efficiently form colonies than A2780 populations (60±4% on average compared with 23±14%, respectively). (**d**) In addition, A2780cis populations were found to grow tumours significantly slower than A2780 populations. Histological analysis demonstrated an ovarian cancer-like pathology. (**e**): CD10^−^/ALDH^−^, (**f**): CD10^−^/ALDH^+^, (**g**): CD10^+^/ALDH^−^), with no consistent differences between the populations. (**h**) All three populations were plated as single cells and assessed for their relationship to one another via SCAD assay. (**i**) This analysis was summarised and modelled and indicated the absence of an obvious (potency-based) apex CSC in this network. Instead, it appears that both CD10^−^/ALDH^+^ and CD10^+^/ALDH^−^ CSCs are now focussed on production of the cisplatin-resistant CD10^−^/ALDH^−^ component. (Population mean comparison using analysis of variance test. Data shown are mean±S.D. of *n*⩾3 mice. Flow cytometric statistics presented as bar charts, NS=not significant, *P-value<0.05; ** *P*-value<0.01, ***P-value<0.001)

The A2780cis parent cell line has been cisplatin adapted by long-term exposure to increasing doses of cisplatin, which, it is recommended, should be added to the cells every 2–3 passages. A2780cis parent cells were grown in the absence of cisplatin to avoid passage to passage alterations to the hierarchy induced by addition of the drug. As described in detail in the next section, this had no effect on cisplatin tolerance in A2780cis cells. In line with the data observed for 96 h cisplatin-treated A2780 cells (Figures 3d and f), A2780cis parent cells were found to contain only three CD10/ALDH populations, CD10^+^/ALDH^+^ CSCs being undetectable (Figure 4a). All CSC populations were again validated by successful generation of tumours from <100 cells in xenograft assays (Figures 4b–g). Although some differences were observed between CD10^−^/ALDH^−^ and CD10^−^/ALDH^+^ populations (first sight of tumour growth, time from injection to endpoint), there was no significant difference in latency period (Figure 4b). Notably, xenograft formation was significantly slower in A2780cis populations compared with A2780 populations (Figure 4d). Additionally, A2780cis cells were found to form colonies significantly slower than A2780 cells, despite A2780cis cells being more efficient at forming colonies (Figure 4c). On average, it took a single A2780cis cell 5–6.5 weeks to grow into colonies of 5 × 10^6^ cells. In contrast, single-plated A2780 cells took 4–4.5 weeks to form colonies containing similar cell numbers. SCAD assays demonstrated an altered hierarchy relationship, with no obvious (potency-based) apex CSC (Figures 4h and i). Specifically, the entire cis-hierarchy is now focussed upon self-renewal and production of the CD10^−^/ALDH^−^ cisplatin-resistant population. As such, it appears that the effects of 96 h cisplatin treatment of A2780 cells are hard-wired, perhaps by longer-term exposure, even when cisplatin is withdrawn for several months.

### A2780cis CD10^−^/ALDH^−^ CSCs display adaptive cisplatin resistance

**Figure 5:**
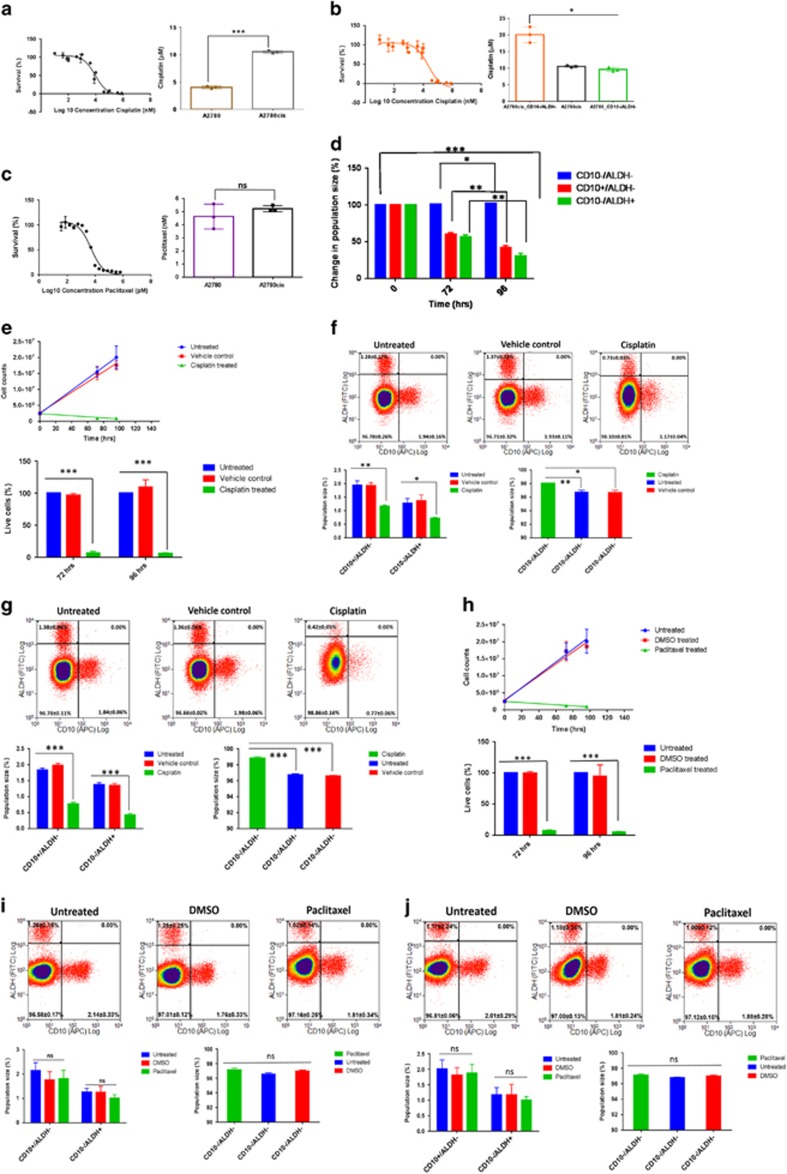
CD10^−^/ALDH^−^ CSCs display adaptive cisplatin resistance and paclitaxel sensitivity. To further elucidate the relationship between the CD10/ALDH network and chemoresistance, (**a**) cisplatin and (**b**) paclitaxel IC_50_s were established. A2780cis cells were found to be more resistant to cisplatin than A2780 cells (**a**: A2780cis IC_50_=11±0.4 *μ*M; *n*=3). A cisplatin dose–response curve indicated that this appears to be due to acquired cisplatin resistance of CD10^−^/ALDH^−^ CSCs (**b**: IC_50_=20.1±0.4 *μ*M; *n*=3). In contrast, A2780 and A2780cis cells were found to be similarly sensitive to paclitaxel (**c**: A2780cis IC_50_=5.2±0.2 *μ*M). A2780cis cells were treated with cisplatin for 72 (**e** and **f**) and 96 (**e** and **g**) hours. Although untreated and DMSO vehicle-treated control cells were unaffected, cisplatin treatment resulted in a proportional increase in the CD10^−^/ALDH^−^ population, as the other populations showed substantial cell death (**d**–**g**). (**h**–**i**) Seventy-two and (**h** and **j**) 96 h 4.6 nM paclitaxel treatment resulted in substantial cell death (**h**), which affected all three populations uniformly (**h**–**j**). All data were confirmed by statistical analysis (bar charts, unpaired Student’s *t*-test, mean population size (%)±S.D. of *n*=3, NS=not significant, ***P-value<0.0001). Together, these data indicate that CD10^−^/ALDH^−^ CSCs possess inherent and acquired cisplatin-resistance mechanisms. Again, the entire ovCSC network is highly sensitive to paclitaxel

As described above, the A2780cis parent cell line was cultured in the absence of cisplatin for several months to avoid inconsistent growing conditions. Subsequently, A2780cis cells were found to have retained cisplatin tolerance (IC_50_=11±0.4 *μ*M, Figure 5a), in line with our previous description.^13^ This indicates that prolonged exposure to cisplatin results in a hard-wired tolerance that is no longer cisplatin dependent. Interestingly, A2780cis parent cells were found to remain highly sensitive to paclitaxel (Figure 5c).

Parent A2780cis cells were treated with their 48 h 11 *μ*m cisplatin IC_50_ (Figures 5d–g), continuing this treatment for 72 and 96 h, and the relative effect on the three CSC populations was assessed by flow cytometry (Figures 5f and g). The results demonstrated that there is substantial cell death following cisplatin treatment (Figures 5d and e). Flow cytometric analysis demonstrated that cisplatin treatment diminished the cisCD10^+^/ALDH^−^ and cisCD10^−^/ALDH^+^ populations relative to untreated and vehicle-treated controls, while the cisCD10^−^/ALDH^−^ population increased in size (Figures 5d–g). This adaptive cisplatin resistance was confirmed by dose–response analysis to be the specific property of cisCD10^−^/ALDH^−^ CSCs (Figure 5b). In isolation, cisCD10^−^/ALDH^−^ CSCs were found to be more cisplatin tolerant than A2780cis parent cells, which is suggestive of proapoptotic paracrine signalling by the other populations (Figure 5b). This is evidence for a continued dominant role for the CD10^−^/ALDH^−^ CSC population, which can further develop inherent cisplatin tolerance through adaptation. Treatment of A2780cis parent cells with their paclitaxel IC_50_ (Figure 5c) resulted in substantial cell death at 72 and 96 h (Figures 5h–j), which affected all three CSC populations uniformly (Figures 5i and j). As such, while it appears that the inherent cisplatin-resistance mechanism seen in CD10^−^/ALDH^−^ CSCs is hard-wired in cisCD10^−^/ALDH^−^ CSCs, the latter population displays additional adaptive cisplatin-resistance mechanisms. The demonstration that all CSC populations are highly sensitive to paclitaxel may have important clinical implications.

### Specific enhanced MDR and DDR mechanisms are associated with inherent and adaptive CD10^−^/ALDH^−^ cisplatin resistance

**Figure 6:**
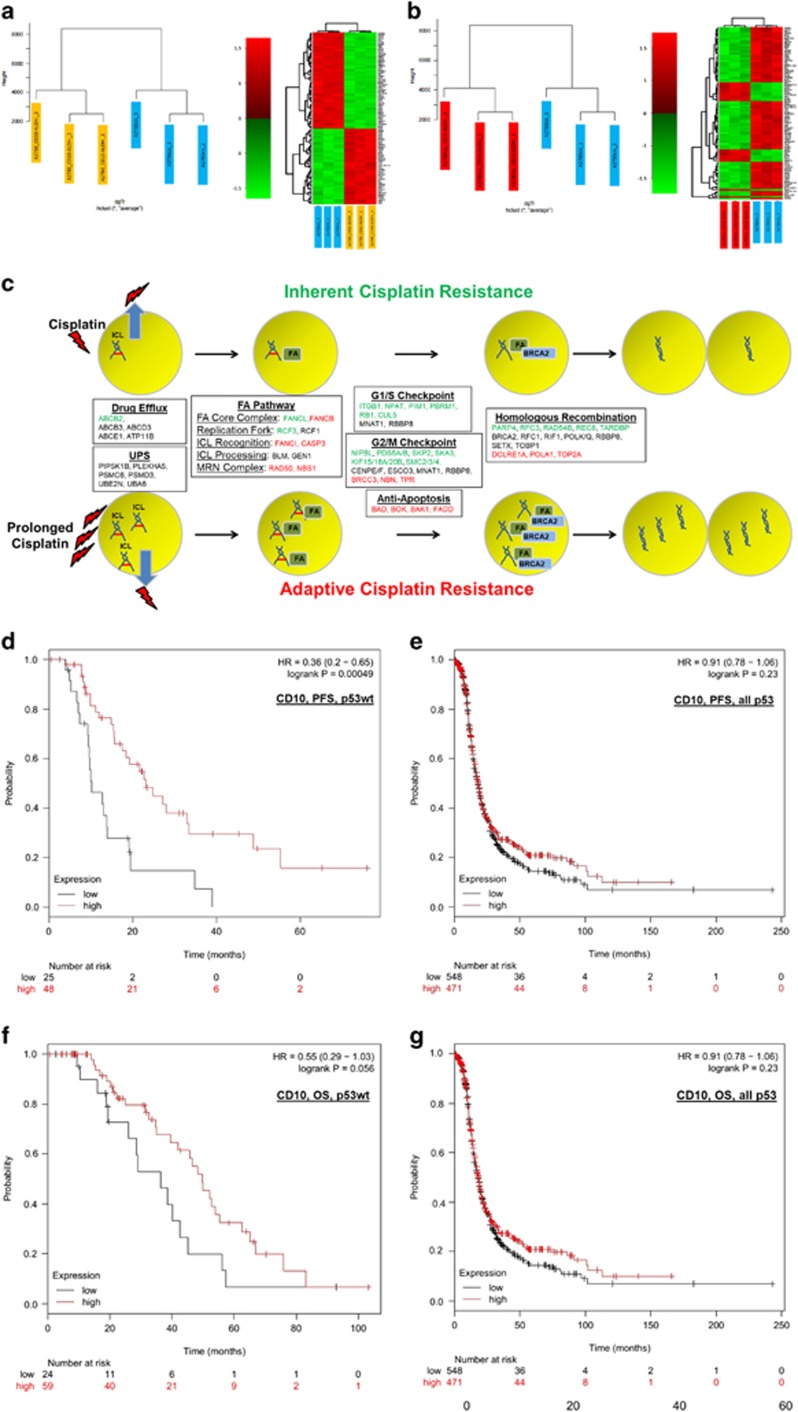

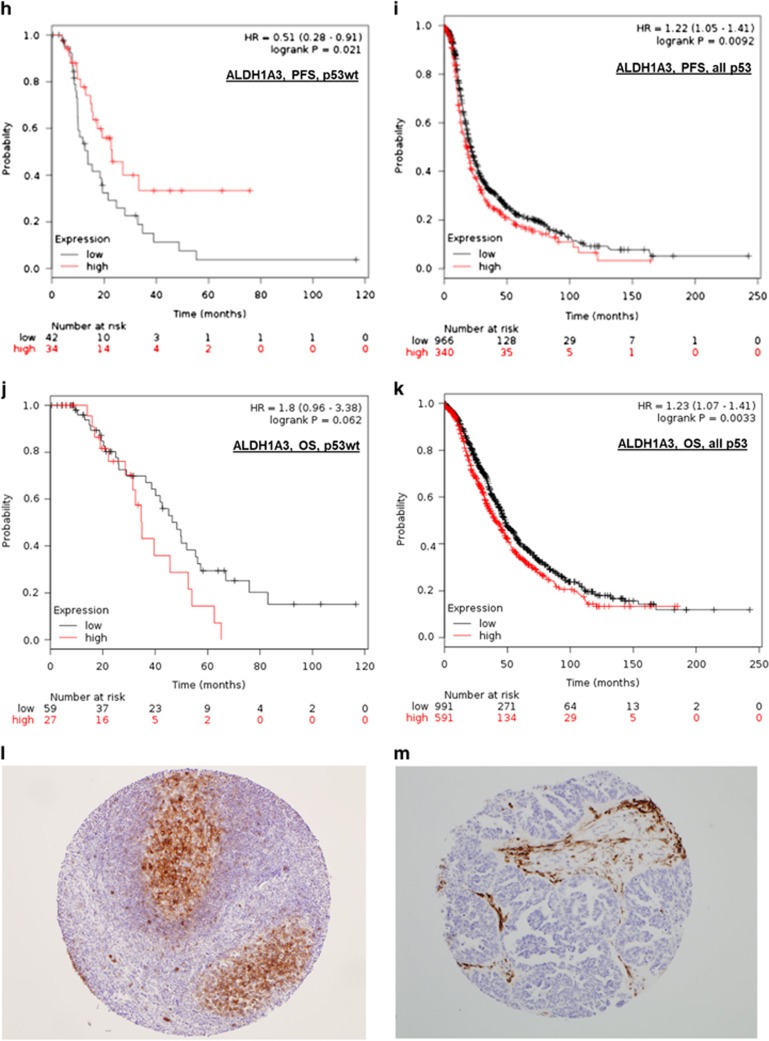
Inherent and adaptive cisplatin resistance in CD10^−^/ALDH^−^ CSCs is associated with enhanced MDR and DDR mechanisms. The molecular mechanisms associated with CD10^−^/ALDH^−^ CSC inherent and adaptive cisplatin resistance were identified using whole-genome gene expression array analysis. (**a** and **b**) Biological replicates of each cell type were found to cluster well. The full genelists and molecular relationships identified by the online tool ‘DAVID’ are shown in Supplementary Data 4. Selected molecular relationships for inherent and adaptive cisplatin resistance are detailed in Table 1. The majority of these genes relate to specific, inherent- and adaptive-resistance MDR and DDR mechanisms. As illustrated ((**c**): inherent genes in green, adaptive genes in red, common genes in black font), these relate to drug efflux (MDR), UPS, the FA pathway (DDR), cell cycle checkpoints, antiapoptosis, and homologous recombination. These data indicate a mechanism where cisplatin resistance of CD10^−^/ALDH^−^ CSCs is facilitated by an enhanced ability to efflux and repair cisplatin-induced ICLs, which is further enhanced by prolonged drug exposure(**d**–**k**). To assess its clinical relevance, CD10/ALDH expression was assessed using the online resource tool Kaplan–Meier Plotter (kmplot.com), which facilitates filtered analysis across a large number of published studies. In each case, the numbers of patients within the cohort who had high or low expression of the protein is shown in red and black, respectively. Statistical significance is indicated as logrank *P* (cutoff *P*<0.05) as well as relative risk of progressive disease (PFS curves) or death (OS curves) as hazard ratio (HR: cutoff <0.77 or >1.3). For each set of analyses, data are shown for p53wt samples only (‘p53wt’) or all p53 types (‘all p53’). This analysis indicated that CD10 negativity was a predictor of poor PFS (**d**–**e**, *P*=0.00049, HR=0.36), with a trend towards worse OS (**f**–**g**, *P*=0.056, HR=0.55), but only in cases of serous carcinoma patients with p53wt disease. Low expression of ALDH1A3 was also found to be a predictor of reduced PFS (**h**, *P*=0.021, HR=0.51) but not OS in p53 wildtype (**j**, *P*=0.062, HR=1.8) or in either PFS (**i**, *P*=0.0092, HR=1.22) or OS (**j**, *P*=0.0033, HR=1.23) when all p53 types were considered together. As a further validation, CD10 expression was assessed in a TMA (Supplementary Data 8) containing germinal centres of (**l**) tonsil positive control and (**m**) ovarian cancer patient tumour samples prepared by our group. CD10 expression was negative in the epithelium of all tumour samples (**m**). In conclusion, CD10/ALDH negativity/low expression may have some utility as prognostic indicators but further validation is required

The molecular mechanisms associated with inherent and adaptive cisplatin resistance of CD10^−^/ALDH^−^ CSCs were characterised using whole-genome gene expression arrays. The complete set of analysis is described in Supplementary data 3 and summarised in Figure 6 and Table 1. Comparison of (A) CD10^−^/ALDH^−^ CSCs with the A2780 parent cell line and (B) cisCD10^−^/ALDH^−^ CSCs with the A2780cis parent cell line established respective genelists of 1772 (inherent; Supplementary Data 4) and 919 (adapted; Supplementary Data 6) differentially expressed genes. Analysis of these genelists via the online bioinformatics resource DAVID highlighted several cisplatin-resistance-related mechanisms (Inherent: Supplementary Data 5; Adaptive: Supplementary Data 7), which are summarised in Figure 6c. In summary, cisplatin resistance is characterised by inherent-specific, adaptive-specific and common expression of MDR and DDR genes, as illustrated in Figure 6c. Specifically, the data suggest a mechanism where (A) cisplatin is effluxed from the cell to reduce ICL formation, (B) ICLs are recognised and processed by specific components of the FA and UPS pathways, (C) time for repair is facilitated through G_1_/S and G_2_/M cell cycle checkpoints and antiapoptosis mechanisms and (D) return to proliferation is facilitated following BRCA2-driven homologous recombination (Figure 6c). We note the expression of an inherent-resistance mechanism (Figure 6c, green text) by cells that have never experienced cisplatin treatment. Notably, adaptive resistance (Figure 6c, red text) is associated with specific expression of genes associated with the FA pathway, particularly the key genes FANCB and FANCI, and further upregulation of BRCA2. In parallel, while the cell cycle checkpoint mechanism is lost in adaptive cisplatin resistance, an antiapoptotic mechanism is suggested by downregulation of proapototic genes. We propose that these increases in the FA pathway and BRCA2 indicate an increased ability to recognise, process and repair ICLs, which is associated with the increased cisplatin tolerance of these cells (Figure 6c). This prolonged cell cycle mechanism is likely to explain the longer time of xenograft tumour formation observed in A2780cis cells (Figures 4c and d). Taken together, these data identify these CSCs ability to self-heal as a potential clinical target, as discussed in detail later.

### Low expression of CD10 is compatible with ovarian cancer patient samples

The data described above suggest that low CD10 expression may be associated with poor outcome (response to platinum) in some ovarian cancer patients. Although low marker expression is not clinically ideal, it was important to demonstrate that high expression of CD10 was not associated with poor outcome in ovarian cancer patients. CD10 expression in ovarian cancer (endometrioid and serous) patient samples was assessed using the online resource tool Kaplan–Meier Plotter, which facilitates filtered analysis across a large number of publically available data sets. In each case, data are shown for p53 wild type (‘p53wt’) or all p53 types (‘all p53’). Low CD10 expression was found to be associated with worse progression-free survival (PFS, *P*=0.00049), with a trend towards worse overall survival (OS, *P*=0.056) but only in patients whose p53 status was wild type (*n*=83, Figures 6d–g). The ALDEFLOUR protocol used to detect ALDH expression in our studies is known to mark several members of the ALDH family. Of the several ALDH proteins available on Kaplan–Meier Plotter, low expression of only ALDH1A3 was found to be associated with worse PFS (*P*=0.021) again in only p53wt patients (Figures 6h–k). We note that low expression of ALDH1A3 is associated with (statistically significant but modest hazard ratio (HR)) improved PFS (*P*=0.0092, HR=1.22) and OS (*P*=0.0033, HR=1.23) when all patients are considered. Further assessing the biomarker utility of the gene signature, all inherent cisplatin-resistance-specific genes listed in Table 1 were individually assessed using Kaplan–Meier Plotter. Supporting the clinical relevance of the inherent cisplatin-resistance mechanism identified, the majority (~68%) of these genes were found to be statistically significant predictors of PFS, 17 genes with HRs of ⩾1.3 (TOPBP1, EYA4, USP1, UACA, TPR, SMC4, SMC3, SLK, SKA3, RFC3, RFC1, PDS5B, PBRM1, KIF20B, ITGB1, FANCL, CENPF; Supplementary Data 8). As a further validation, we assessed CD10 expression in a tissue microarray (TMA) consisting of 46 high-grade (serous) ovarian cancer patients (demographics detailed in Supplementary Data 8). Although strong CD10 expression was observed in positive control tissue (Figure 6l) contained in the TMA, the epithelium of all tumours was negative or showed at most focal non-specific staining (Figure 6m). As such, while further analysis and validation is required, low CD10 expression is consistent with specific sets of ovarian cancer patient samples.

## Discussion

In this study, we used a single-cell analysis approach to build a new ovCSC hierarchy model, which facilitated testing of the respective chemoresistance of each CSC type. Our data indicated that, in this model, chemoresistance is specific to both CSC type and drug type. Historically, chemoresistance has been thought of as a general property of CSCs. Our data indicates that, within the tumour, different populations of CSC can be responsible for resistance to specific chemotherapy drugs. In concordance with this, we have found that only ALDH^+^ (but not ALDH^-^) CSCs are cisplatin resistant in three models of lung cancer.^14^ These are the first studies of their kind and require examination in other malignancies. However, these data have potential implications for clinical targeting of CSCs, which are now discussed.

Our study has implications for our understanding of CSC theory and hierarchy organisation. It is evident that CSCs can be organised as a non-linear network where the apex CSC can be produced by a less potent progenitor and that progenitor CSCs can produce one another. This indicates that the apex CSC can be regenerated by a lower potency progenitor and may thus be a less attractive clinical target in some types of malignancy.^3^ It is also evident that a CSC hierarchy may contain chemosensitive and chemoresistant components, which are dramatically altered by chemotherapy treatment. Pretreatment and posttreatment, CSCs can express multiple specific chemoresistance mechanisms. These changes persist after removal of cisplatin, suggesting that they are hard-wired by chemotherapy. As we have discussed in our recent review,^8^ this presents a clinical consideration where CSC hierarchies can be dynamic rather than fixed targets, which must be modelled to identify appropriate clinical targets. Where appropriate clinical targets can be identified by approaches such as those described herein, there is great hope for the translation of CSC targeting to the clinic. To facilitate clinical translation, it is important that the principles identified herein are tested in other malignancies to identify malignancy-specific CSC targets.

In terms of ovCSCs specifically, a previous consensus model informed by the current literature indicates that ovarian cancer is characterised by a hierarchical network of CSC types.^15^ The authors highlight that the ‘XXX’ marker for the ‘XXX^+^?’/ALDH^−^/CD133^−^/CD44^−^/CD117^−^ apex CSC must be identified. The model we have identified is evidence for CD10^−^/ALDH^−^ CSCs (also CD133^−^/CD44^−^/CD117^−^, Supplementary Data 1) as the consensus model apex CSC. However, as our model contrasts the Burgos–Ojeda model by placing a different cell (CD10^+^/ALDH^+^) at the apex of the hierarchy, clearly more work is required to understand the full disease. It is particularly important for ovarian cancer, which is recognised as a collection of different diseases, that highly specific analysis of CSCs associated with specific malignant traits displayed by specific types of disease is undertaken.^3^

Finally, our data offer a clinical consideration where, in future, it may be possible to exploit CSC analysis to identify groups of patients that are likely to respond to specific chemotherapy regimens, which has been highly successful in breast cancer treatment.^25, 26, 27^ The data presented in this study are evidence for the presence of a subdivision of ovarian cancer that may respond better to paclitaxel than to cisplatin. Together with our similar work in lung cancer,^14^ the data presented herein support the hypothesis that CSC analysis may facilitate future triaging of patients into specific chemotherapy response groups. It is important to follow this study with a similar approach directed towards elucidation of the relationship between CSC hierarchies and chemoresistance in other cancer models.

Although low and high expression of BRCA1 is associated with cisplatin sensitivity and resistance, respectively,^19, 20^ our highlighting of a link between high BRCA2 expression and cisplatin resistance has rarely been described.^21, 22, 23^ Reactivation of BRCA2 via secondary mutations is linked to cisplatin resistance due to restored DDR in inherited ovarian cancer.^22, 23^ Antisense targeting of BRCA2 in cancer cells with functional HR has been shown to enhance cisplatin response.^24^ Our results here suggest a potential for clinical targeting of FA/BRCA2 pathway overexpression in specific platinum-resistant patients. Selective targeting of an intact FA/BRCA2 pathway is an emerging approach to cancer therapy.^25, 26, 27, 28^ Several FA pathway inhibitors have been developed and shown to be successful in addressing cisplatin resistance.^29, 30^ Although more development is required, FA targeting clearly represents a potential clinical strategy.

Together, our data support a hypothesis where a specific drug may be appropriate for a specific patient group but associated with some negative effects for another patient group, a concept that has been demonstrated in breast cancer. Targeting specific DDR mechanisms may offer hope for future translation to the clinic. Finally, our data indicate an attractive clinical approach, where, in specific patients, targeting of CD10^−^/ALDH^−^ CSCs at initial treatment may be sufficient to compromise the development/progression of recurrent ovarian disease.

## Materials and methods

### Cell culture and drug treatments

Cell lines (A2780/A2780cis) and culturing are as previously described.^13^ Where stated, A2780cis cells were cultured in the absence of cisplatin. Cisplatin (Hospira, Maidenhead, UK) was donated by the Aseptic Compounding Unit, St. James’s Hospital, Dublin, Ireland. Paclitaxel powder (Sigma, Wicklow, Ireland) was prepared in dimethyl sulphoxide (DMSO). For IC_50_ analysis, 5000 cells/well in a 96-well plate were treated with concentration ranges of 0.01–800 *μ*M and 9 mg/ml sodium chloride (Cisplatin) and 0.0375–625 nM (Paclitaxel), using vehicle controls of 1 mg/ml mannitol (Cisplatin) and DMSO (Paclitaxel). After 48 h, cell viability was assessed using the Cell Counting Kit-8 (CCK-8, Sigma). IC_50_ values were calculated using GraphPad Prism (La Jolla, CA, USA).

### Histological and transcriptomic analysis of patient samples

A TMA was prepared from a cohort composed of 46 patients with high-grade serous carcinoma of tubal/ovarian origin. Cases were represented as triplicate cores and 39 cases contained at least 2 assessable cores. A list of the demographics for each patient is available in Supplementary Data 8. Sections were cut at 4 *μ*m intervals. Germinal centres of tonsil were used as a positive control. CD10 (DAKO (Stockport, UK) clone 56C5, code M7308) staining was carried on a Ventana BenchMark XT (Tucson, AZ, USA) at a 1 : 40 dilution using CC2 antigen retrieval. Detection was achieved using the Ventana Optiview Kit. An additional publically available data set was interrogated using the online resource Kaplan–Meier Plotter (kmplot.com).

### Statistical analysis

Statistical analysis was performed using GraphPad Prism 6. The unpaired Student’s *t*-test was used to compare two population means. A one-way ANOVA was carried to compare three or more population means. A *P*-value of <0.05 was considered statistically significant. Data are represented as mean and S.D. of *n*=3. Kaplan–Meier analysis was used to calculate PFS and OS from the TMA. Patient survival time was compared by the log-rank test. All significance testing was two-sided, where a *P*-value⩽0.05 was considered to be statistically significant.

### Additional methods

Validation of CSCs, via xenograft and SCAD assay, and flow cytometric analysis are described in Supplementary Data 1.

## Figures and Tables

**Table 1:** Selected genes specifically expressed by CD10^−^/ALDH^−^ CSCs, as identified by DAVID analysis (Full list in Supplementary Data 4-7)

**Process**	**Genes**	***P*-value**
*Inherent Cisplatin Resistance*		
Response to DNA-damage	ATAD5, BLM, FANCL, GEN1, RAD54B, RIF1, SMG1, SP100, SLK, ATRX, BRCA2, ESCO1, EYA4, MNAT1, MAPK26, PARP4, POLK, POLQ, RFC1, RFC3, RBBP8, SETX, SMC3, SMC5, SMC6, TOBP1, USP1, UBE2N, UACA	2.7 × 10^−4^
DNA-damage	BLM, FANCL, GEN1, RAD54B, SMG1, SLK, ATRX, BRCA2, ESCO1, EYA4, MNAT1, PARP4, POLK, POLQ, RFC1, RFC3, RBBP8, SETX, SMC3, SMC5, SMC6, TOBP1, USP1, UBE2N	3.2 × 10^−4^
DNA Repair	BLM, NIPBL, PDS5A, PDS5B, RAD54B, REC8, SKP2, TARDBP, TTK, BRCA2, CENPE, CENPF, SKA3, CUL5, ITGB1, KIF15, KIF18A, KIF20B, MNAT1, NPAT, PIM1, PBRM1, RB1, RBBP8, SMC2, SMC3, SMC4, TDRD1	2.8 × 10^−3^
Cell Cycle Checkpoint	BLM, TTK, CENPE, CENPF, DLG1, RB1, RBBP8, TPR	4.9 × 10^−2^

*Adaptive Cisplatin Resistance*		
Response to DNA Damage Stimulus	ATAD5, BRCC3, BLM, DCLRE1A, FANCB, FANCI, GEN1, RAD50, RAD54B, RIF1, SMG1, SLK, BRCA2, CASP3, ESCO1, MNAT1, NBN, POLK, POLA1, POLQ, RCF1, RBBP8, SETX, SMC3, SMC5, SMC6, TOP2A, TOPBP1, USP1, UACA	1.2 × 10^−8^
DNA Repair	BRCC3, BLM, DCLRE1A, FANCB, FANCI, GEN1, RAD50, RAD54B, SMG1, SLK, BRCA2, ESCO1, MNAT1, NBN, POLK, POLA1, POLQ, RCF1, RBBP8, SETX, SMC3, SMC5, SMC6, TOP2A, TOPBP1, USP1	1.1 × 10^−8^
Double-strand Break Repair	BRCC3, BLM, RAD50, RAD54B, BRCA2, NBN, POLA1, SETX	5.5 × 10^−4^
Nucleotide-Excision Repair	DCLRE1A, SLK, BRCA2, MNAT1, RFC1	3.8 × 10^−2^
Cell Cycle Checkpoint	BRCC3, BLM, TTK, CENPE, CENPF, NBN, RBBP8, TPR	5.2 × 10^−2^
DNA Replication	XRN1, POLA1, TOPO2A, TOPO2B	2.3 × 10^−3^
