# Supplementary material for: CD10−/ALDH− cells are the sole cisplatin-resistant component of a novel ovarian cancer stem cell hierarchy
Source: Cell Death Dis. 2017 Oct 19;8(10):e3128–. doi: 10.1038/cddis.2017.379 (PMC5680566; doi:10.1038/cddis.2017.379)
Supplement: Supplementary Data 1 [file cddis2017379x1.docx]

**Ffrench et al 2017.**

**CD10^-^/ALDH^-^ Cells are the Sole Cisplatin-Resistant Component**

**of a Novel Ovarian Cancer Stem Cell Hierarchy**

**Supplementary Data 1. Identification and Validation of the CD10 and ALDH Markers.**

Contents

**Part 1**: Identification and validation of ALDH as a stemness marker in the A2780 model.

**Part 2**: Identification of CD10 and validation of the CD10/ALDH stemness markers in the A2780 model.

**Part 1: Identification and validation of ALDH as a stemness marker in the A2780 model.**

**1.1: Results**

Herein we describe the screening of the A2780 and A2780cis models for cancer stemness markers. The study began by screening for stemness properties (Hoechst dye efflux and Aldehyde Dehydrogenase [ALDH] activity) and markers (CD44, CD117, CD133 and CXCR4) that were present in both the cisplatin-sensitive treatment-naïve cancer model A2780 and its long-term cisplatin-adapted counterpart A2780cis. A2780/A2780cis is a highly characterised cisplatin-sensitive/adapted model that was originally derived from a treatment-naïve ovarian cancer patient of unknown histology/pathology. Both cell types were found to contain positive and negative populations of stemness marker ALDH activity (Table S1, Figure 1A-B). A2780 cells contained a CD133^+^ population, while neither cell type contained CD44, CD117 or CXCR4 positive populations, or possessed efflux ability as measured by Hoechst dye efflux assay (Table S1, Figure S1.1). ALDH positive (ALDH^+^) and negative (ALDH^-^) populations were isolated from A2780 cells via FACS and assessed for validity as CSCs using xenograft and single-cell asymmetric division (SCAD) assays. In this SCAD assay, ALDH^+^ and ALDH^-^ cells isolated via FACS were plated as single cells and allowed to develop into colonies. Subsequently, colonies were assessed via flow cytometry for the presence of ALDH+ and/or ALDH- cells. ALDH^+^ and ALDH^-^ cells were shown to generate xenograft tumours with high efficiency in immune-compromised mice (Figure 1C-E, Figures S1.2 & S1.3). All ALDH^+^ but only some ALDH^-^ (designated ALDH^-A^) cells tested were found to produce ALDH^+^ and ALDH^-^ populations when plated as single cells and allowed to form colonies (SCAD assay, Figure 1F-G). However, some ALDH^-^ cells (designated ALDH^-B^) produced only ALDH^-^ cells via SCAD assay (Figure 1H). When isolated via FACS these ALDH^-B^ cells were confirmed to produce xenograft tumours (Figure 1C). Similar SCAD data was observed for A2780cis cells (Figure S1.4).This data suggested that ALDH^-B^ cells are CSCs whose differentiation product is not ALDH^+^ cells. Further elucidation of ALDH^-A^ CSCs required identification of a specific ALDH^-A^ marker.

**1.2 Materials and Methods**

1.2.1: Flow Cytometry and FACS analysis

Cells were dissociated using 1mM EDTA and 1 x 10^6^ cells prepared for analysis. Following specific staining procedures, samples were analysed on a CyAN ADP flow cytometer (Beckman Coulter) or sorted using a FACSAria Fusion Cell Sorter (BD Bioscience). ALDH analysis was carried our using the ALDEFLOUR™ assay (Stem Cell Technologies), using Diethylaminobenzaldehyde (DEAB) to inhibit the reaction as a negative control, all as per manufacturer’s instructions. The Hoechst side population assay was carried out using Hoechst 33342 dye (5µg/ml, H342, Sigma), using efflux inhibitor Verapmil (50µM) as a negative control. For cell surface protein staining, the following antibodies and isotype controls were used: CD10 (LT10, Immunotools & 1F8, Abcam), CD44 (F10-44-2 & X5563, Abcam), CD117 (104D2 & ICIGG1, Abcam), CD133 (293C3 & IS6-11E5.11, Miltenyi), CXCR4 (12G5 & 20102, R&D Systems). Identical concentrations were used for both primary antibody and isotype controls, which acted as a non-specific staining control. A sample with cells only was used in each assay to account for autofluorescence. Propidium Iodide (PI) was used as a dead cell stain (0.5µg/ml). A 99.5% confidence interval (mean ± 3x Standard Deviation) was used to identify populations as reliably detectable, regardless of their sizes.

1.2.2: Xenograft Tumourigenicity Assay

All animal research work reported in this article was carried out in the Comparative Medicine Unit, Trinity College Dublin, a Health Products Regulatory Authority (HPRA) approved establishment that operates in accordance with Directive 2010/63/EU and its Irish transposition S.I No 543 of 2012. Trinity College Dublin complies with the ‘Council for International Organizations of Medical Sciences’ (CIOMS), International Guiding Principles for Biomedical Research Involving Animals, and all laws, regulations and policies governing the care and use of laboratory animals in the jurisdiction in which the research is being conducted. 7-9 week old female NOD.CB17-Prkdcscid/NCrHsd (NOD.SCID, Harlan) and NOD.CB17-Prkdcscid/NcrCrl mice (Charles River) were used for *in vivo* xenograft experiments. Cells were administered via subcutaneous hind limb injection in a 100µl 4:1 mixture of Ham’s F12 media (Lonza):Matrigel® (Corning) to n=4 animals per experiment. Tumours of any size were considered for analysis, where a scientific end-point of maximum 1cm diameter tumour measured by callipers was used, in line with UKCCCR guidelines. Harvested tumours were fixed in 10% neutral buffered formalin, embedded in paraffin wax, sectioned, H+E stained, and assessed by two pathologists (JOL, BD, RD). Statistical analysis was carried out using Graphpad Prism 6. The Log-Rank (Mantel-Cox) test was performed to compare the survival curves. The unpaired student’s t-test was used to compare two population means. A one-way ANOVA was carried to compare three or more population means. A p-value of < 0.05 was considered statistically significant.

1.2.3: Validation of CSCs

CSCs were validated using xenograft tumour assays, as described in Supplementary Data 1, and SCAD assays. For the SCAD assays, subpopulations were sorted to >98% purity by FACS, which was then immediately verified by re-analysis. Single cells were plated into 60 wells of a 96-well plate and allowed to expand over time, transferring to larger flasks as appropriate. This was repeated for 3 independent experiments. Clones totalling 5 x 10^6^ cells were stained for CD10 and ALDH expression and analysed via flow cytometry.

All other methods are described in the Materials and Methods section of the main manuscript.

**Table S1**. A summary of the results of the A2780 and A2780cis stem cell marker screen.

| Population Size (%) | | | | | | |
| --- | --- | --- | --- | --- | --- | --- |
| Model | **CD44** | **CD117** | **CD133** | **CXCR4** | **Hoechst** | **ALDH** |
| A2780 | 0.343± 0.140 | 0.017± 0.006 | 0.063± 0.015 | 0.050± 0.036 | 0.010± 0.010 | 0.203± 0.031 |
| A2780cis | 1.447± 1.098 | 0.017± 0.012 | 0.007± 0.006 | 0.120± 0.131 | 0.003± 0.006 | 0.773± 0.076 |


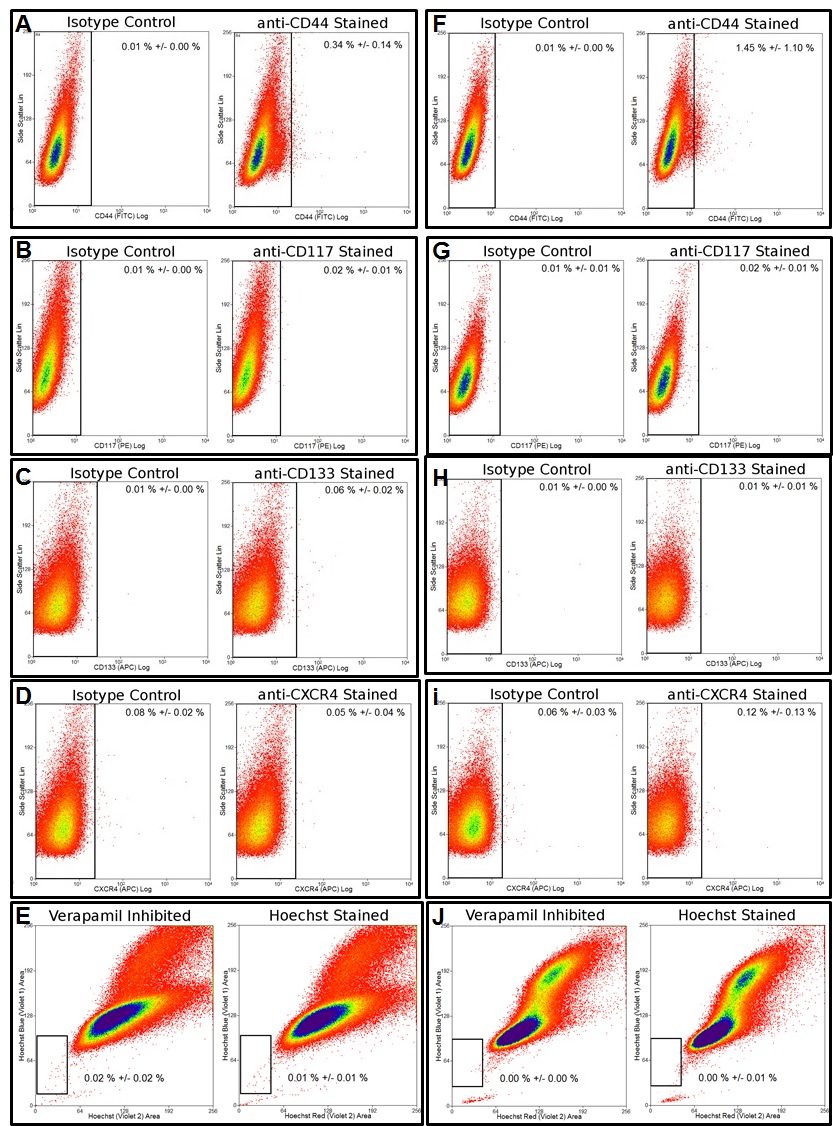


**Figure S1.1. The A2780 and A2780cis models contain no CD44, CD117, CD133, CXCR4 or Hoechst efflux positive populations.**

The treatment naïve A2780 and cisplatin-adapted A2780cis ovarian cancer cell line models were assessed for the expression of a panel of CSC markers. The images show representative flow cytometry charts for each antibody and an appropriate isotype control. A2780 cells were found to contain a small CD133 population (C) but no CD44 (A), CD117 (B), or CXCR4 (D) populations. A2780 cells did not display an ability to efflux Hoechst dye when compared to controls treated with efflux inhibitor verapamil (E). Similarly, A2780cis cells were found to contain no CD44 (F), CD117 (G), CD133 (H) or CXCR4 (i) populations or Hoechst dye efflux ability (J).


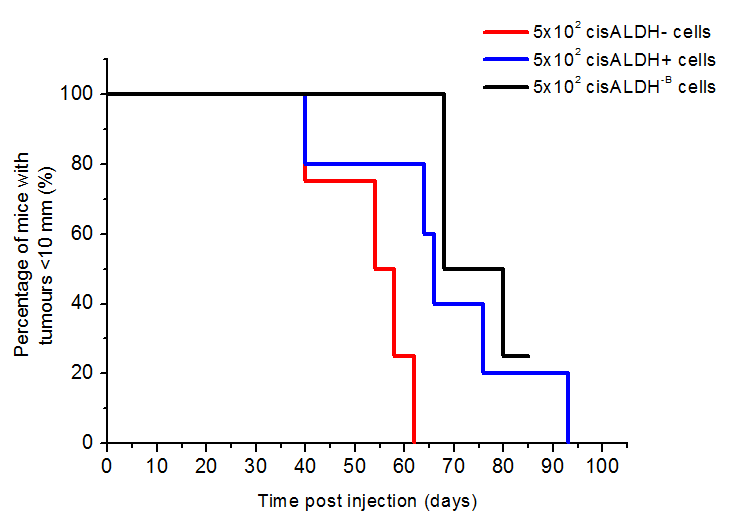


**Figure S1.2. ALDH^+^ and ALDH^-^ cells isolated from A2780cis cells can efficiently generate xenograft tumours.**

ALDH^+^ and ALDH^-^ cells were isolated from the A2780cis parent cell line. Each cell type was injected into the hind-limb of immune-compromised mice, and generation of xenograft tumours monitored over time. No statistical difference was observed between cisADLH^+^ and cisALDH^-^ cells in terms of latency (p=0.1972). cisALDH^-B^ cells were found to have a significantly longer latency period than the cisALDH^-^ cells (p=0.015) but not cisALDH^+^ cells (p=0.534).


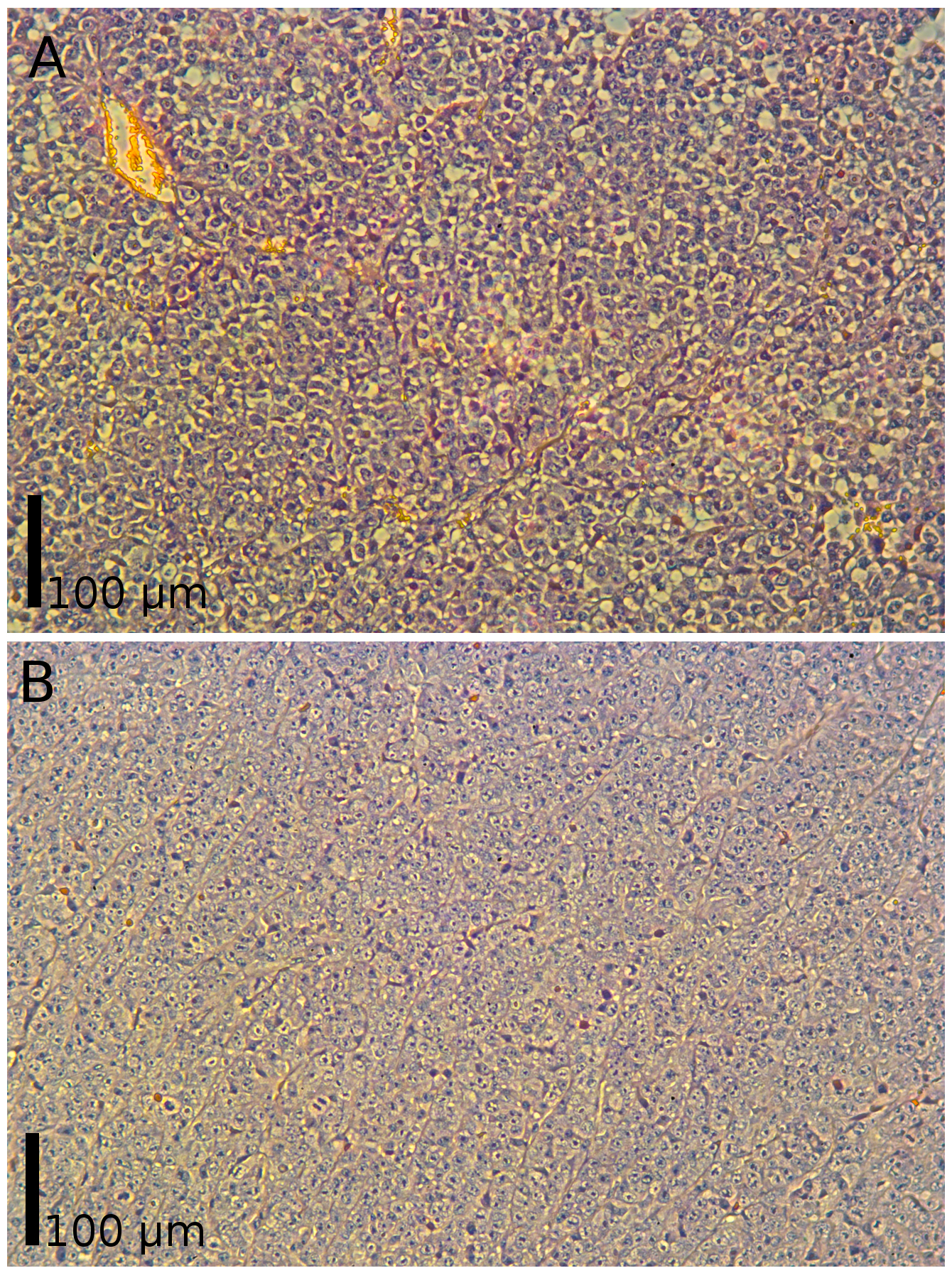


**Figure S1.3. Pathological analysis of A278cis ALDH^+^ and ALDH^-^ cell xenograft tumours.**

Tumours generated during the experiments described in Figure S1.2 were harvested, prepared on to slides and haematoxylin and eosin (H+E) stained for pathological analysis. The images (100X) show the pathology of xenografts generated from ALDH^+^ (A) and ALDH^-^ (B) cells.


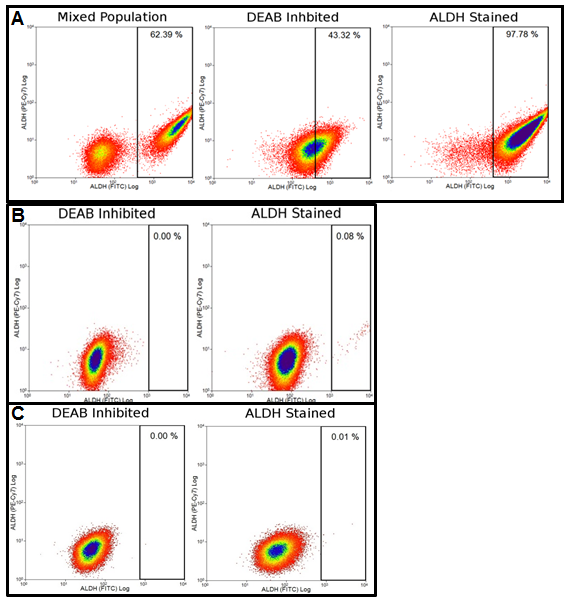


**Figure S1.4. A2780cis ALDH^+^ cells self-renew and differentiate in SCAD assay.**

Single ALDH^+^ and ALDH^-^ A2780cis cells were plated as single cells and allowed to expand over time before being harvested and assessed for ADLH activity (using ALDH inhibitor DEAB as a negative control for non-specific binding) via flow cytometry (SCAD assay). The ALDEFLUOR assay could not resolve the ALDH^+^ population from the DEAB control when the sample contained >80% ALDH^+^ cells. To address this, a ‘mixed population’ control sample containing a mix of the ‘test’ cells with known negative cells (A2780) was included in the analysis and the mid-point between the two populations used to set the gating threshold (A). ALDH^+^ (A) and ALDH^-A^ (B) cells were found to produce both ALDH^+^ and ALDH^-^ cells. No clones tested could be confidently determined as ALDH^-B^. However, some ALDH^-^ clones were deemed ‘Unclassified’, as they did not meet the criteria for either ALDH^-A^ or ALDH^-B^ (C). Together with the xenograft analysis shown in Figure S1.2 above, these data indicate that ALDH^-A^ cells are a CSC population that can generate ALDH^+^ cells during differentiation. In contrast, ALDH^-B^ cells are a validated CSC that cannot produce ALDH^+^ cells during differentiation.

**Part 2: Identification of CD10 and validation of the CD10/ALDH stemness markers in the A2780 model.**

2.1 Introduction: The ALDH^-A^ marker problem

Data described in Figures 1 and 2 of the main manuscript and in part 1 above describe how A2780 and A2780cis cells were found to contain multiple ALDH subpopulations. ALDH^+^ cells were found to be capable of producing ALDH^+^ and ALDH^-^ cells in SCAD assays. Some, ALDH^-^ cells, termed ALDH^-B^, produced only ALDH^-^ cells in SCAD assays. The colonies resulting from these SCAD assays contained only ALDH^-B^ cells. Other ALDH^-^ cells, termed ALDH^-A^, produced both ALDH^+^ and ALDH^-^ cells in SCAD assays. The colonies resulting from these SCAD assays were a mixture of ALDH^+^, ALDH^-A^ and ALDH^-B^ cells. ALDH^+^ cells could easily be removed from these samples, resulting in mixed ALDH^-A^ + ALDH^-B^ samples. However, further analysis required identification of a specific ALDH^-A^ marker to isolate pure ALDH^-A^ clones for analysis. To achieve this, whole genome gene expression analysis was performed on mixed ALDH^-A^ + ALDH^-B^ samples compared to pure ALDH^-B^ samples (Figure S2.1). This subtractive approach permitted the identification of potential ALDH^-A^ markers.

**2.2 Materials and Methods**

2.2.1: Gene Array Analysis

Whole genome gene expression array analysis was performed with 15ug cRNA using the GeneChip® Human 2.0 ST arrays (Affymetrix). RNA was isolated using the mirVANA kit (Life Technologies) and assessed for quality and quantity using the Agilent 2000 Bioanalyser. cRNA was prepared for analysis using the WT Expression and Terminal Labelling Kits (Ambion/Life Technologies). GeneChips were washed using the Fluidics Station 450 and scanned using the GeneChip Scanner 3000 (both Affymetrix). All protocols as per manufacturer’s instructions.

Array quality control was carried out as per the Affymetrix quality assessment white paper [1], using the Affymetrix expression console software. All data were analysed using Bioconductor software libraries ([www.bioconductor.com](http://www.bioconductor.com/)). The ‘oligo’ package was used to import Affymetrix CEL file data [2]. These data were then used to compute robust multichip average (RMA) expression values [3]. The ‘made4’ package was used for visualisation of RMA expression values and RankProd was used to identify expression differences between gene chips [4-5]. Gene array data for the individual CD10/ALDH populations required adjustment for a batch-effect. This was corrected by removing non-coding RNAs from the data, normalisation of individual comparisons instead of normalising all data together, and removal of the batch effect using Limma [6]. For this analysis, Hierarchical clustering was carried out using the Manhattan distance method and probes annotated using the latest Biomart version [7] based on Ensembl genes [8]. Cut-off values used were ±2 fold change and an adjusted p-value (false discovery rate: FDR) of <0.05. Functional annotation was achieved using the online resource DAVID [9-10].

All other methods are described in the Materials and Methods section of the main manuscript.

**2.3 Results**

ALDH^-^ cells were sorted to >98% purity via FACS, plated as single cells and allowed to form colonies over time. Colonies were subsequently harvested and tested for the presence of ALDH^+^ and ALDH^-^ cells via flow cytometry. Colonies containing only ALDH^-^ cells were designated as ‘ALDH^-B^‘. To identify potential ALDH^-A^ markers, pure populations of ALDH^-^ cells (containing ALDH^-A^ and ALDH^-B^ cells) were compared to pure ALDH^-B^ populations via gene expression microarray analysis. Gene array analysis was carried out as described in the methods section above (Illustrated in Figure S1.1), with samples clustering well under analysis (Figure S2.2). From the genelist generated (Supplementary Data 2), 7 cell surface markers were selected as potential markers within the top 20 genes (Table S2.1). No commercial antibody was available for transmembrane protein 2 (TMEM2). Therefore it had to be omitted from the screening panel. EPHA3, EPHA7, ZPLD1 and FLRT3 expression was not detected in cells from any of the samples via flow cytometry. As such, these 4 potential markers were eliminated from consideration. Finally, VCAN showed staining in the ALDH^-B^ clones (22.46%±4.05%), but was undetectable in the ALDH^-A^ clones (0.24%±0.15%; isotype control 0.23%±0.05 %; Figure S2.3). Therefore, VCAN was eliminated as a suitable marker. The remaining marker CD10 (MME) was the only cell surface protein fulfilling the criteria of an optimal ALDH^-A^ marker (Table S2.2, Figure S2.4). First, a substantial CD10^+^ subpopulation was detected in the parent A2780 cell line (69.46%±2.01%, Figure S2.4). Second, a significantly greater expression was detected in the ALDH^-A^ clones (88.6%±8.74%; p‑value =0.0017, Figure S2.4). Third, no expression was detected in ALDH^‑B^ clones (0.21%±0.07 %; isotype control 0.28%±0.03%, Figure S2.4).

CD10 was thus chosen as the ALDH^-A^ marker for further analysis in double stains with ALDH using flow cytometry. The data presented in Figure S2.5 demonstrates that CD10 and ALDH staining are compatible. This was a critical checkpoint in the experimental design of this project. CD10/ALDH staining identified 4 subpopulations within the A2780 cell line: CD10^-^/ALDH^-^, CD10^+^/ALDH^-^, CD10^-^/ALDH^+^ and CD10^+/^ALDH^+^. CD10^-^/ALDH^-^ and CD10^+^/ALDH^-^ are two major populations, which make up 39.41%±0.21% and 60.33%±0.21%, respectively. CD10^-^/ALDH^+^ and CD10^+^/ALDH^+^ are two minor populations, which make up 0.06%±0.01% and 0.2%±0.01%, respectively.


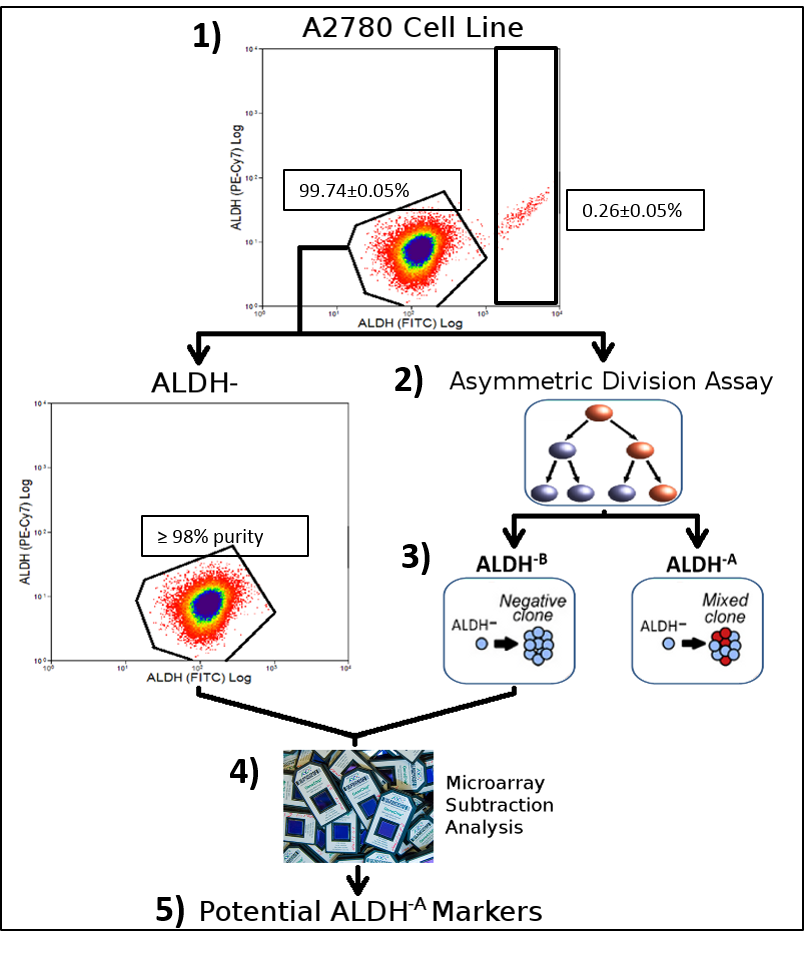


**Figure S2.1****: A subtraction approach was used to identify the putative ALDH^-A^ marker.**

This figure gives an overview of the subtraction approach used to identify the putative ALDH^-A^ marker. 1) The cell line A2780 contains a small ALDH+ population of less than 1% (0.26%± 0.05%). For the subtraction approach, ALDH^-^ cells were sorted to purity (≥ 98%) from the parent cell line A2780 via FACS. 2) Using the SCAD assay, those sorted cells were plated as single cells into 96-well plates and allowed to grow into colonies. 3) Colonies were re‑tested for ALDH expression to classify clones as ALDH^-A^, which produce a mix of ALDH^-^ and ALDH^+^, or ALDH^-B^, which can only make themselves. 4) Pure populations of ALDH^-^ and ALDH^-B^ clones were brought forward to microarray analysis. 5) The ALDH^-B^ gene signature was subtracted from the ALDH^-^ gene signature. The resulting gene list allowed us to identify the potential ALDH^‑A^ markers.


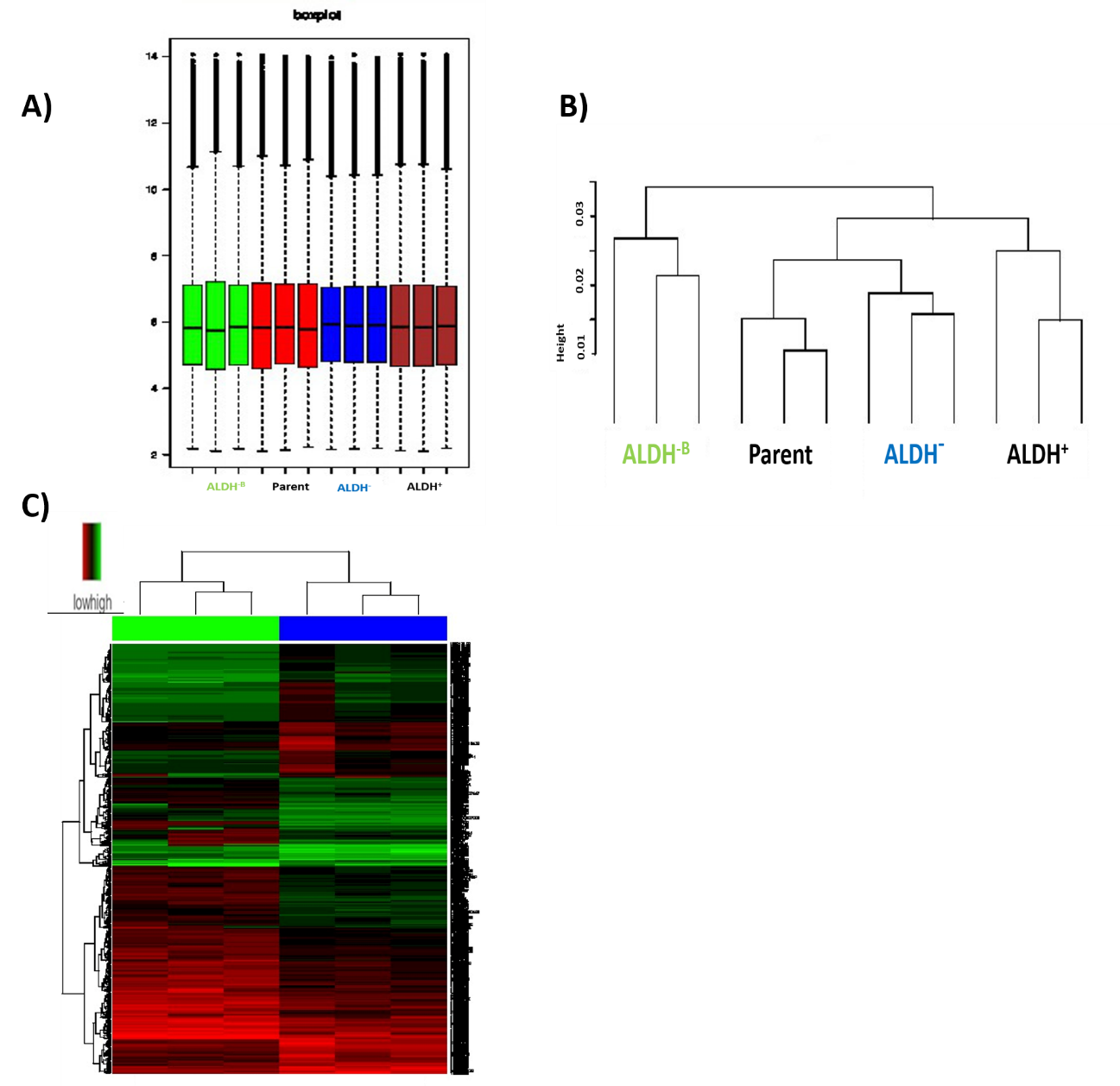


**Figure S2.2: Gene level differential expression analysis of ALDH^-^ and ALDH^-B^ samples.**

In this work a subtraction approach was used to identify a potential ALDH^‑A^ marker. The image shows data from three individual biological replicates for each treatment used for gene level differential expression analysis. In A and B, ALDH^-B^ samples are highlighted in green and ALDH^-^ samples are highlighted in blue. A) The boxplot shows the intensities of each array post-normalization. It shows a normal distribution of signal intensities with little variation within treatment groups and between treatment groups. B) The data shows strong separation of the samples into their treatment groups by hierarchical clustering. C) The heat map shows gene expression of ALDH^-^ samples compared to ALDH^-^**^B^** samples. The key in the upper left of the panel shows the relative differential expression corresponding to the colours in the heat map.

**Table S2.1: Potential ALDH^-A^ marker identified through microarray analysis**


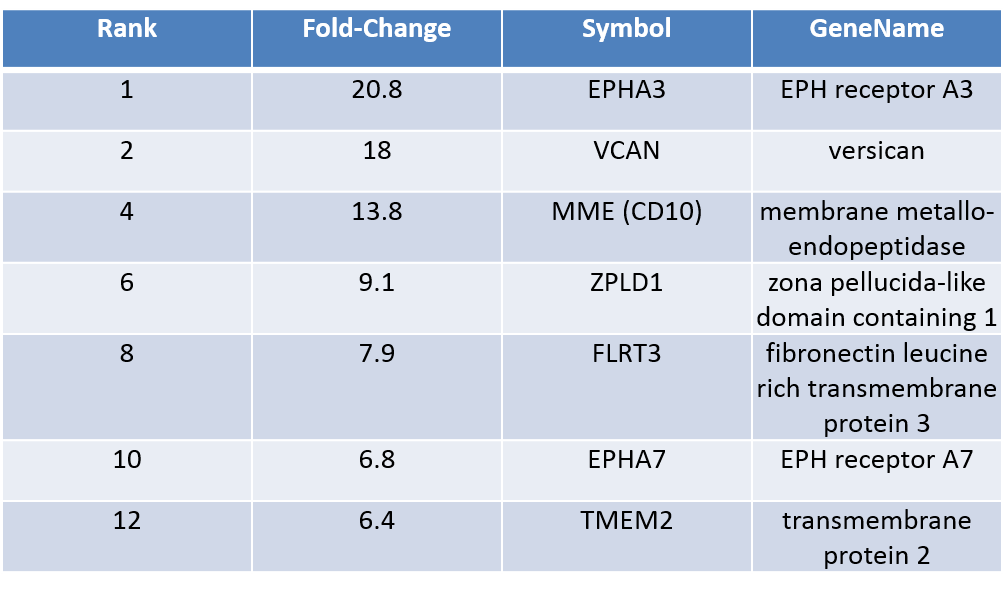


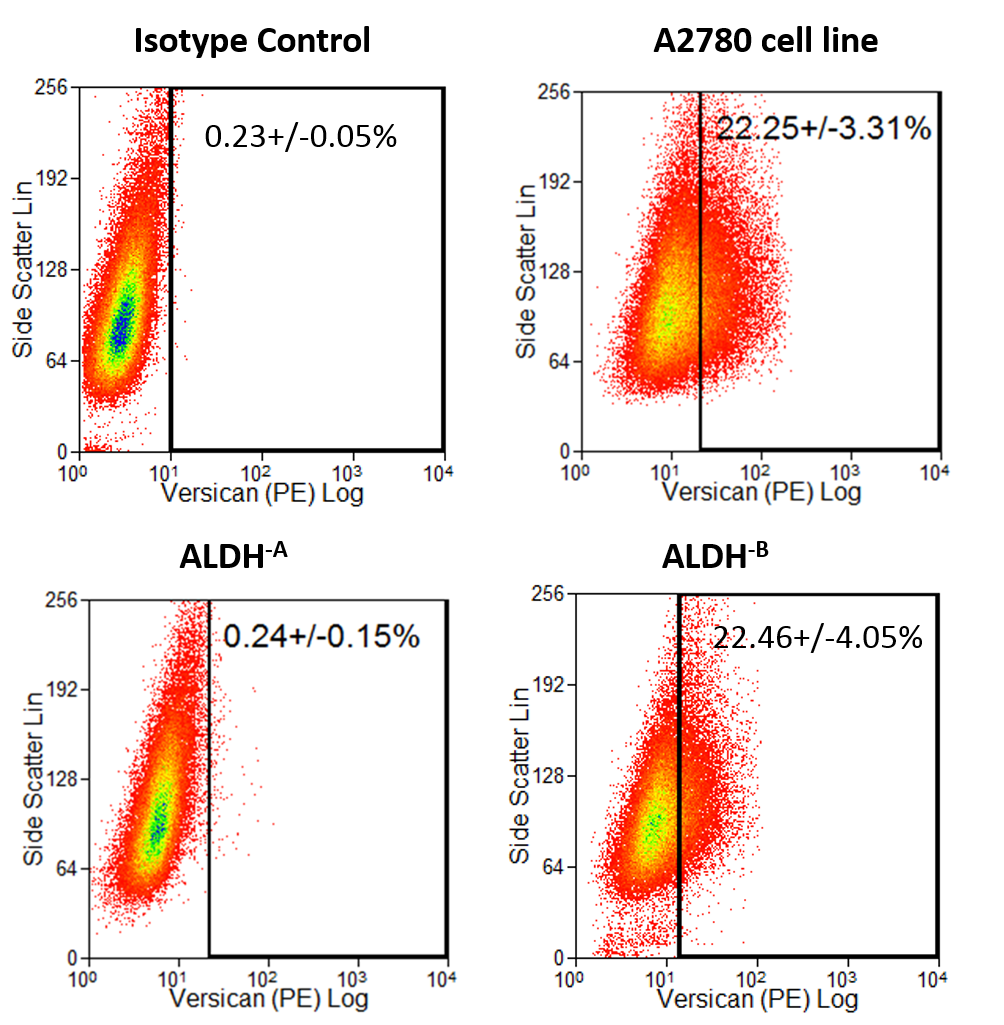


**Figure S2.3: VCAN does not fulfil the ALDH^-A^ marker criteria.**

The flow cytometry graphs show the expression of VCAN in the parent cell line A2780, the ALDH^‑A^ clones and the ALDH^‑B^ clones. The isotype control was used to control for non-specific staining caused by the primary antibody. The x-axis represents the fluorescence intensity of VCAN and the y-axis represents the side scatter, which is proportional to cell granularity. The data is shown as mean population size (%) ± standard deviation of n=3 experiments. 22.25%±3.31% of the parent cell line A2780 showed VCAN expression. 24.46%±4.05% of the ALDH^-B^ clones showed VCAN expression, whereas no protein expression was detectable in the ALDH^‑A^ clones (0.24%± 0.15%; isotype control 0.23%±0.05%). VCAN did not fulfil all three all ALDH^-A^ marker criteria. It was therefore ruled out as a potential ALDH^-A^ marker.

**Table S2.2: CD10 fulfils the ALDH^-A^ marker criteria**


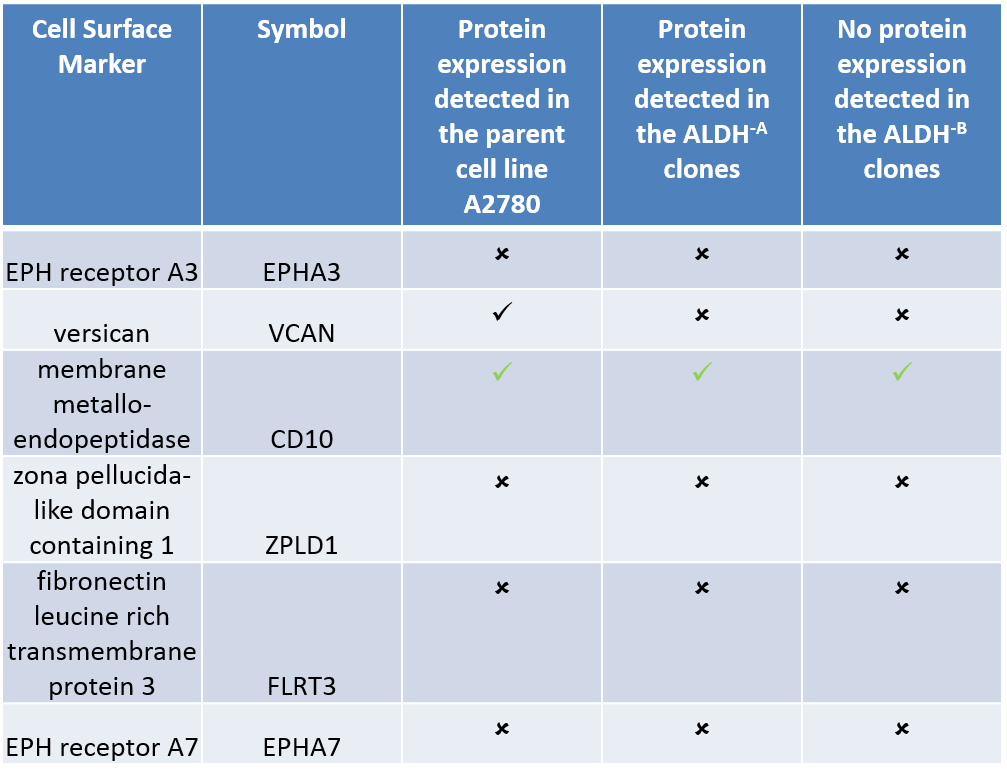


**
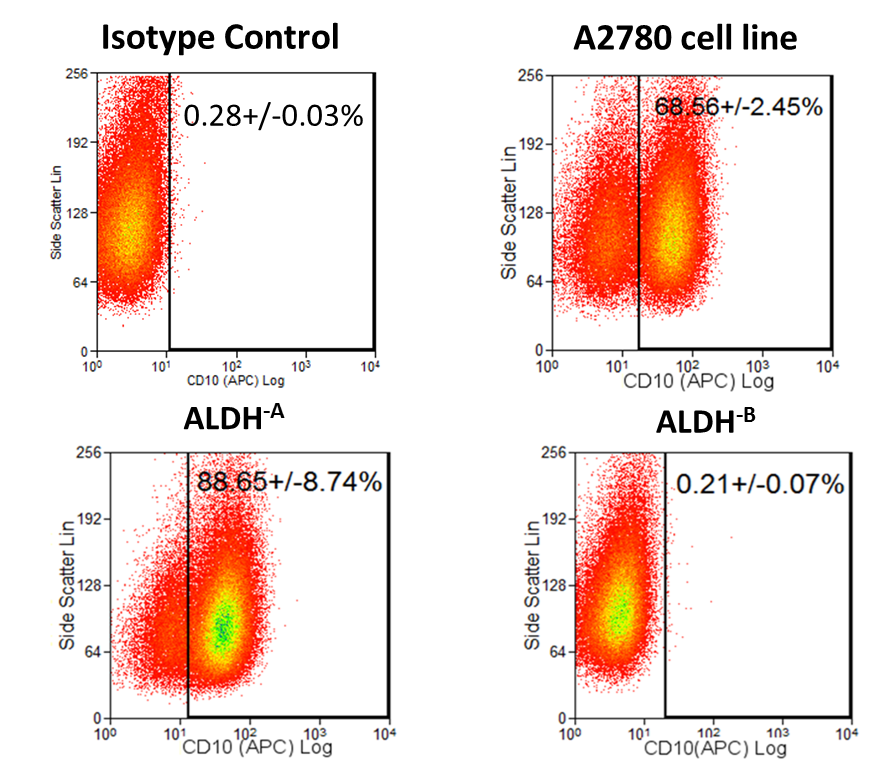
**

**Figure S2.4: CD10 was identified as the best potential ALDH^-A^ marker.**

The flow cytometry graphs show the expression of CD10 in the parent cell line A2780, the ALDH^‑A^ clones and the ALDH^‑B^ clones with respect to the isotype control. The x-axis represents the fluorescence intensity of CD10 and the y-axis represents the side scatter, which is proportional to cell granularity. The data is shown as mean population size (%) ± standard deviation of n=3 experiments. 68.56%±2.45% of the parent A2780 population showed CD10 expression. 88.65%±8.74 % of the ALDH^-A^ clones showed CD10 expression. No protein expression was detectable in the ALDH^‑B^ clones (0.21% ±0.07%; isotype control 0.28%±0.03%). CD10 fulfilled all three all ALDH^-A^ marker criteria. It was therefore validated as an ALDH^-A^ marker.


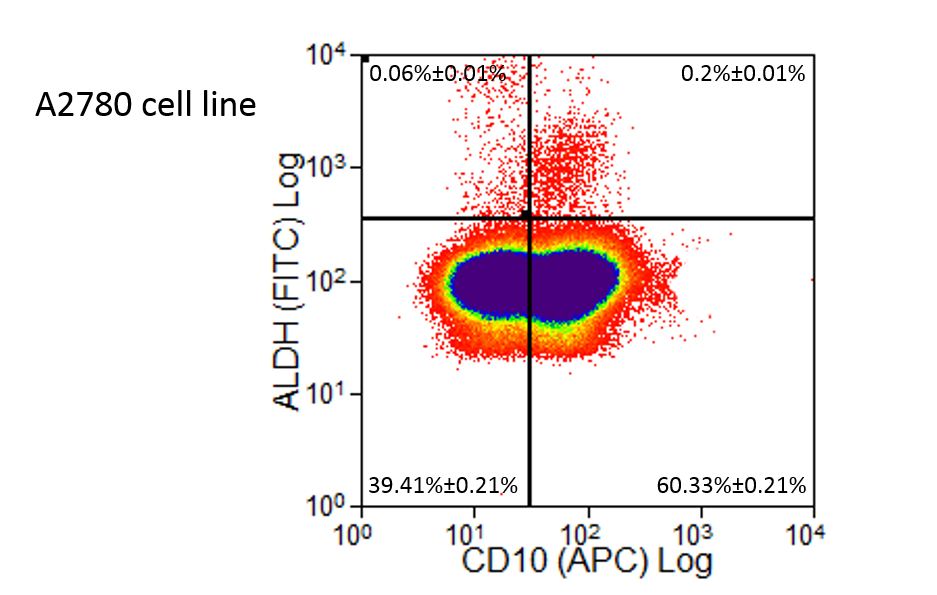


**Figure S2.5: CD10/ALDH double staining identifies four subpopulations in the A2780 cell line.**

This flow cytometry graph shows the four subpopulations identified by CD10/ALDH double staining. The x-axis represents the fluorescence intensity of CD10 and the y-axis represents the fluorescence intensity of ALDH. The mean population size (%) ± standard deviation of n=3 experiments identifies two major populations; CD10^-^/ALDH^-^ (39.41%±0.21%) and CD10^+^/ALDH^-^ (60.33%±0.21 %) and two minor populations; CD10^-^/ALDH^+^ (0.06%±0.01%) and CD10^+^/ALDH^+^ (0.2%±0.01 %).

**References**

1. Gentleman R. Bioinformatics and computational biology solutions using R and bioconductor. In: Gentleman R editor. Statistics for biology and health. Springer. 2005

2. Carvalho BS, Irizarry RA. A framework for oligonucleotide microarray preprocessing. Bioinformatics. 2010, 26(19): 2363–2367.

3. Irizarry RA, Hobbs B, Collin F, Beazer-Barclay YD, Antonellis KJ, Scherf U, Speed TP. Exploration, normalization, and summaries of high density oligonucleotide array probe level data. Biostatistics. 2003, 4 (2), pp. 249–264.

4. Culhane AC, Thioulouse J, Perrière G, Higgins DG. MADE4: an R package for multivariate analysis of gene expression data. Bioinformatics 2005, 21(11): 2789–2790.

5. Breitling R, Armengaud P, Amtmann A, Herzyk P. Rank Products: A simple, yet powerful, new method to detect differentially regulated genes in replicated microarray experiments. FEBS Lett. 2004, 573(1-3): 83–92.

6. Ritchie ME, Phipson B, Di W, Hu Y, Law CW, Shi W, Smyth GK. (2015): limma powers differential expression analyses for RNA-sequencing and microarray studies. Nuc Acids Res. 2015, 43 (7): e47.

7. Smedley D, Haider S, Durinck S, Pandini L, Provero P, Allen J. et al. The BioMart community portal: an innovative alternative to large, centralized data repositories. Nuc Acids Res. 2015, 43 (W1): W589-98.

8. Kinsella RJ, Kähäri A, Haider S, Zamora J, Proctor G, Spudich G. et al. Ensembl BioMarts: a hub for data retrieval across taxonomic space. Database. 2011, 2011: bar030.

9. Huang DW, Sherman BT, Lempicki RA. Bioinformatics enrichment tools: paths toward the comprehensive functional analysis of large gene lists. Nucl Acids Research. 2009a, 37 (1): 1–13.

10. Huang DW, Sherman BT, Lempicki RA. Systematic and integrative analysis of large gene lists using DAVID bioinformatics resources. Nature Protocols. 2009b, 4(1): 44–57.
