# Supplementary material for: CD10−/ALDH− cells are the sole cisplatin-resistant component of a novel ovarian cancer stem cell hierarchy
Source: Cell Death Dis. 2017 Oct 19;8(10):e3128–. doi: 10.1038/cddis.2017.379 (PMC5680566; doi:10.1038/cddis.2017.379)
Supplement: Supplementary Data 2 [file cddis2017379x2.docx]

**Ffrench et al 2017.**

**CD10^-^/ALDH^-^ Cells are the Sole Cisplatin-Resistant Component**

**of a Novel Ovarian Cancer Stem Cell Hierarchy**

**Supplementary Data 2. A Detailed Description of the Gene Array Analysis from which the Cisplatin-Resistance Molecular Mechanisms were identified.**

Contents

**Part 1:** Enhanced MDR & DDR Mechanisms are Associated with Inherent CD10^-^/ALDH^-^ Cisplatin-Resistance

**Part 2:** Enhanced DDR and Anti-Apoptosis Mechanisms are Associated with Adaptive CD10^-^/ALDH^-^ Cisplatin-Resistance

**Part 1. Enhanced MDR & DDR Mechanisms are Associated with Inherent CD10^-^/ALDH^-^ Cisplatin-Resistance**

**1.1: Introduction**

Whole-genome gene array analysis was used to identify the inherent (treatment-naïve) cisplatin-resistance mechanism expressed by CD10^-^/ALDH^-^ CSCs derived from the A2780 cell line. These data are summarised in the main manuscript and described in detail here.

**1.2: Results**

We next characterised the molecular mechanisms associated with inherent cisplatin-resistance of CD10^-^/ALDH^-^ CSCs using whole-genome gene expression array analysis (for methods see Supplementary Data 1). Comparison of CD10^-^/ALDH^-^ cells with A) A2780 parent cells and B) CD10^+^/ALDH^-^ cells identified 235 and 514 differentially expressed genes respectively (Supplementary Data 4). However, when these were analysed for molecular relationships using the online bioinformatics resource DAVID [14], no cisplatin-resistance mechanisms were highlighted, which may reflect our poor understanding of cisplatin-resistance generally (Supplementary Data 6). In contrast, a CD10^-^/ALDH^-^ versus A2780cis parent cell (intrinsic- versus adaptive-resistance) comparison identified a larger number (1772) of differentially expressed genes (Supplementary Data 4), from which a mechanism was highlighted via DAVID analysis (Supplementary Data 6).We note, therefore, that the inclusion of a cisplatin-adapted comparator in our analysis aided in the highlighting of the intrinsic-resistance mechanism. These data are described in detail here and summarised in Figure 6A, C and Table 1. In Figure 6C we propose a model where inherent cisplatin-resistance is associated with a collection of ABC family drug efflux genes, namely TAP1/ABCB2, TAP2/ABCB3, ABCD3 and ABCE1. Subsequently, additional genes associated with recognition and repair of ICLs by the FA and NER pathways, and HR, are upregulated. Notably, this includes increased expression of key HR gene BRCA2, which is in line with our previous description of the A2780 model as BRCA wildtype [15]. This FA/BRCA2 mechanism appears to be facilitated by the specific expression of UPS (PIP5K1B, PLEKHA5, PSMC6, PSMD3, UBE2N and UBA6) and downregulation of pro-apoptosis (FADD, BAK1, BOK and BAD) genes. This suggests a mechanism where A) cisplatin is effluxed from the cell to reduce ICL formation, B) ICLs are recognised and processed by specific components of the FA pathway, C) time for repair is facilitated through G_1_/S and G_2_/M cell cycle checkpoints and anti-apoptosis mechanisms, and D) return to proliferation is facilitated following BRCA2-driven HR (Figure 6C). We note that these mechanisms are expressed by cells that have never experienced cisplatin treatment. We also note that the specific expression of G_2_/M checkpoint genes, particularly with respect to the SAC, is in concordance with the paclitaxel-sensitivity of CD10^-^/ALDH^-^ CSCs. As such, the CSC population responsible for inherent, treatment naïve cisplatin-resistance within the CD10/ALDH hierarchy is characterised by high expression of drug efflux and DNA-damage monitoring/repair mechanisms.

**Part 2. Enhanced DDR and Anti-Apoptosis Mechanisms are Associated with Adaptive CD10^-^/ALDH^-^ Cisplatin-Resistance**

**2.1: Introduction**

Whole-genome gene array analysis was used to identify the adaptive (post-treatment) cisplatin-resistance mechanism expressed by CD10^-^/ALDH^-^ CSCs derived from the A2780cis cell line. These data are summarised in the main manuscript and described in detail here.

**2.2: Results**

Molecular mechanisms associated with adaptive cisplatin-resistance were assessed through two comparisons. These data are described in detail here and summarised in Figure 6B-C. The obvious comparison of CD10^-^/ALDH^-^ and cisCD10^-^/ALDH^-^ CSCs resulted in a large genelist of 917 differentially expressed genes (Supplementary Data 5). However, when assessed using DAVID this list did not facilitate modelling of a mechanism, which again may reflect our poor understanding of cisplatin-resistance generally (Supplementary Data 7). In contrast, the comparison of cisCD10^-^/ALDH^-^ cells with A2780cis parent cells identified 919 differentially expressed genes showing a clear cisplatin-resistance mechanism via DAVID analysis (Supplementary Data 7). As illustrated in Figure 6C and detailed in Table 1, these data indicated continued expression of many of the drug efflux, FA pathway, UPS and HR genes, while the inherent-specific expression of many G_1_/S and G_2_/M checkpoint genes was lost. Furthermore, additional, cisplatin-adapted expression of genes associated with the FA pathway, particularly the key genes FANCB and FANCI, and genes related to the G_2_/M checkpoint and HR was observed. Notably, BRCA2 expression was further upregulated in cisplatin-adapted CD10^-^/ALDH^-^ CSCs compared to treatment-naïve CD10^-^/ALDH^-^ CSCs. In parallel, this population downregulates pro-apoptotic genes (BAD, BOK, BAK1, FADD and p53), which suggests an anti-apoptotic mechanism (Supplementary Data 5). While this appears to facilitate DDR in response to cisplatin, it seems likely that continued paclitaxel-sensitivity in these cells is due to the synergistic effect of inherent and adaptive G_2_/M checkpoint genes. We propose that these increases in the FA pathway and BRCA2 indicate an increased ability to recognise, process and repair ICLs, which is associated with the increased cisplatin-tolerance of these cells (Figure 6C). This prolonged cell cycle mechanism is likely to explain the longer time of xenograft tumour formation observed in A2780cis cells (Figure 4C-D). Finally, it is noted that this mechanism was identified despite the fact that the populations being compared (A2780cis parent and cisCD10^-^/ALDH^-^ cells) are 98% similar. We note that there is a large bias in the data towards genes that are uniquely expressed by the 2% (cisCD10^-^/ALDH^-^ and cisCD10^+/^ALDH^-^ cells) population. The transcriptomics data here supports our earlier suggestion of pro-apoptotic paracrine signalling between the 3 cell types in the A2780cis parent cell line. Taken together, these data identify these CSCs ability to self-heal as a potential clinical target, as discussed in detail later.
