# Supplementary material for: CD10−/ALDH− cells are the sole cisplatin-resistant component of a novel ovarian cancer stem cell hierarchy
Source: Cell Death Dis. 2017 Oct 19;8(10):e3128–. doi: 10.1038/cddis.2017.379 (PMC5680566; doi:10.1038/cddis.2017.379)
Supplement: Supplementary Data 8 [file cddis2017379x8.docx]

**Ffrench et al 2017.**

**CD10^-^/ALDH^-^ Cells are the Sole Cisplatin-Resistant Component**

**of a Novel Ovarian Cancer Stem Cell Hierarchy**

**Supplementary Data 8**

**S8.1 Details of Tissue Microarray (TMA)**

All details associated with patient samples that make up the TMA are shown in Supplementary Methods Table 1. Material for the TMA was abotained though the DISCOVARY bioresource. Patients received informed consent, and ethical approval was received from the ‘Joint St James’s Hospital/ Adelaide and Meath Hospital Dublin, incorporating the National Children’s Hospital’, Ethics Committee.

The median age of the population was 59.5 (range 37-86) years, the median progression free survival (PFS) was 23 (range 5-119) months, the median disease free interval (DFI) was 18.5 (range 0-114) months, the median overall survival (OS) was 4845±31 (range 5-125) months. Patient tumours were staged according to the FIGO system. 4/46 (8.7%) were stage 1, 3/46 (6.5%) were stage II, 35/46 (76.1%) were stage III and 4/46 (8.7%) were stage IV. 4/46 (8.7%) were grade 2, and 42/46 (91.3%) were grade 3. Of these, 6/46 (13%) patients underwent suboptimal debulking (>1cm residual disease) and 40/46 (87%) patients underwent optimal surgical debulking (<1cm residual disease). As of July 2015 at the time of analysis, 24/46 (52.2%) were alive and 22/46 (47.8%) had died. 33/46 (71.7%) had recurred.

**Supplementary Table 8.1**. Details of the patients samples contained in the TMA

| TCDOG | Age | Debulking | Stage | Grade | DFI | PFS | OS | Recurred | Status |
| --- | --- | --- | --- | --- | --- | --- | --- | --- | --- |
| 8 | 59 | OPTIMAL | 3 | 3 | 10 | 16 | 43 | Recurred | Dead |
| 15 | 60 | SUBOPTIMAL | 3 | 3 | 11 | 17 | 41 | Recurred | Dead |
| 36 | 86 | SUBOPTIMAL | 3 | 2 | 3 | 7 | 9 | Recurred | Dead |
| 38 | 66 | OPTIMAL | 2 | 3 | 42 | 45 | 124+ | Recurred | Alive |
| 52 | 60 | OPTIMAL | 3 | 2 | 89 | 95 | 125+ | Recurred | Alive |
| 71 | 41 | OPTIMAL | 4 | 3 | 0 | 5 | 7 | Recurred | Dead |
| 75 | 54 | OPTIMAL | 3 | 3 | 14 | 20 | 47 | Recurred | Dead |
| 80 | 63 | OPTIMAL | 2 | 3 | 39 | 46 | 95 | Recurred | Dead |
| 87 | 60 | OPTIMAL | 3 | 3 | 114+ | 119+ | 119+ | No Recurrence | Alive |
| 89 | 44 | OPTIMAL | 3 | 3 | 2 | 7 | 18 | Recurred | Dead |
| 115 | 61 | SUBOPTIMAL | 3 | 3 | 100+ | 105+ | 105+ | No Recurrence | Alive |
| 137 | 51 | OPTIMAL | 3 | 3 | 16 | 21 | 73 | Recurred | Dead |
| 159 | 60 | SUBOPTIMAL | 3 | 3 | 7 | 12 | 23 | Recurred | Dead |
| 165 | 50 | OPTIMAL | 3 | 3 | 51 | 56 | 103+ | Recurred | Alive |
| 172 | 72 | OPTIMAL | 3 | 3 | 2 | 7 | 11 | Recurred | Dead |
| 184 | 57 | SUBOPTIMAL | 4 | 3 | 1 | 5 | 9 | Recurred | Dead |
| 188 | 74 | OPTIMAL | 3 | 3 | 30 | 38 | 75 | Recurred | Dead |
| 189 | 59 | OPTIMAL | 3 | 2 | 18 | 22 | 48 | Recurred | Dead |
| 195 | 44 | OPTIMAL | 3 | 2 | 9 | 13 | 30 | Recurred | Dead |
| 223 | 49 | OPTIMAL | 1 | 3 | 92+ | 97+ | 97+ | No Recurrence | Alive |
| 225 | 52 | OPTIMAL | 3 | 3 | 8 | 14 | 28 | Recurred | Dead |
| 228 | 76 | OPTIMAL | 3 | 3 | - | - | 4 | Recurred | Dead |
| 257 | 61 | OPTIMAL | 1 | 3 | 80+ | 85+ | 85+ | No Recurrence | Alive |
| 260 | 58 | OPTIMAL | 3 | 3 | 19 | 25 | 46 | Recurred | Dead |
| 268 | 64 | OPTIMAL | 1 | 3 | 59+ | 64+ | 64+ | No Recurrence | Alive |
| 277 | 86 | OPTIMAL | 2 | 3 | - | - |  | No Recurrence | Alive |
| 282 | 66 | OPTIMAL | 3 | 3 | 35 | 41 | 82 | Recurred | Alive |
| 287 | 79 | OPTIMAL | 3 | 3 | 54 | 59 | 84+ | Recurred | Alive |
| 303 | 58 | OPTIMAL | 3 | 3 | 67+ | 74+ | 81+ | No Recurrence | Alive |
| 313 | 70 | OPTIMAL | 3 | 3 | 48 | 52 | 79+ | Recurred | Alive |
| 358 | 40 | OPTIMAL | 3 | 3 | - | 23 | 25 | Recurred | Dead |
| 369 | 65 | OPTIMAL | 3 | 3 | - | - | 68+ | Recurred | Alive |
| 373 | 72 | OPTIMAL | 3 | 3 | 15 | 21 | 61 | Recurred | Alive |
| 391 | 59 | OPTIMAL | 3 | 3 | 17 | 22 | 41 | Recurred | Dead |
| 444 | 54 | OPTIMAL | 3 | 3 | 54+ | 60+ | 60+ | No Recurrence | Alive |
| 456 | 37 | OPTIMAL | 3 | 3 | 16 | 21 | 47 | Recurred | Alive |
| 473 | 52 | OPTIMAL | 3 | 3 | 0 | 20+ | 20+ | No Recurrence | Alive |
| 481 | 53 | OPTIMAL | 3 | 3 | 49+ | 54+ | 54+ | No Recurrence | Alive |
| 507 | 56 | OPTIMAL | 3 | 3 | 44+ | 53+ | 53+ | No Recurrence | Alive |
| 515 | 59 | OPTIMAL | 3 | 3 | 2 | 7 | 16 | Recurred | Dead |
| 517 | 66 | OPTIMAL | 3 | 3 | 36 | 43 | 50+ | Recurred | Alive |
| 562 | 68 | OPTIMAL | 1 | 3 | 42+ | 48+ | 48+ | No Recurrence | Alive |
| 574 | 82 | OPTIMAL | 3 | 3 | - | - | 5 | Recurred | Dead |
| 649 | 67 | SUBOPTIMAL | 4 | 3 | - | - | 8 | Recurred | Dead |
| 661 | 57 | OPTIMAL | 4 | 3 | 13 | 19 | 37+ | Recurred | Alive |
| 717 | 43 | OPTIMAL | 3 | 3 | 7+ | 10+ | 10+ | No Recurrence | Alive |
| 8 | 59 | OPTIMAL | 3 | 3 | 10 | 16 | 43 | Recurred | Dead |
| 15 | 60 | SUBOPTIMAL | 3 | 3 | 11 | 17 | 41 | Recurred | Dead |
| 36 | 86 | SUBOPTIMAL | 3 | 2 | 3 | 7 | 9 | Recurred | Dead |

**TCDOG**: Internal identifier; **DFI**: Disease-Free Interval; **PFS**: Progression-Free Survival; **OS**: Overall Survival; **-** indicates progressive disease; + indicates no event at time of follow up.

**S8.2 Clinical Analysis of the Inherent Cisplatin-Resistance Gene Signature**

Details of an inherent cisplatin-resistance gene signature are provided in Table 1 of the main manuscript. Each of these genes was individually assessed for the potential utility as an indicator of reduced Progressive Free Survival (PFS) using the online tool Kaplan-Meier Plotter. These data are shown in the images below. The majority (~68%) of these genes were found to be statistically significant predictors of PFS. Of these, 17 genes were found to have hazard ratios of ≥1.3 (TOPBP1, EYA4, USP1, UACA, TPR, SMC4, SMC3, SLK, SKA3, RFC3, RFC1, PDS5B, PBRM1, KIF20B, ITGB1, FANCL, CENPF).


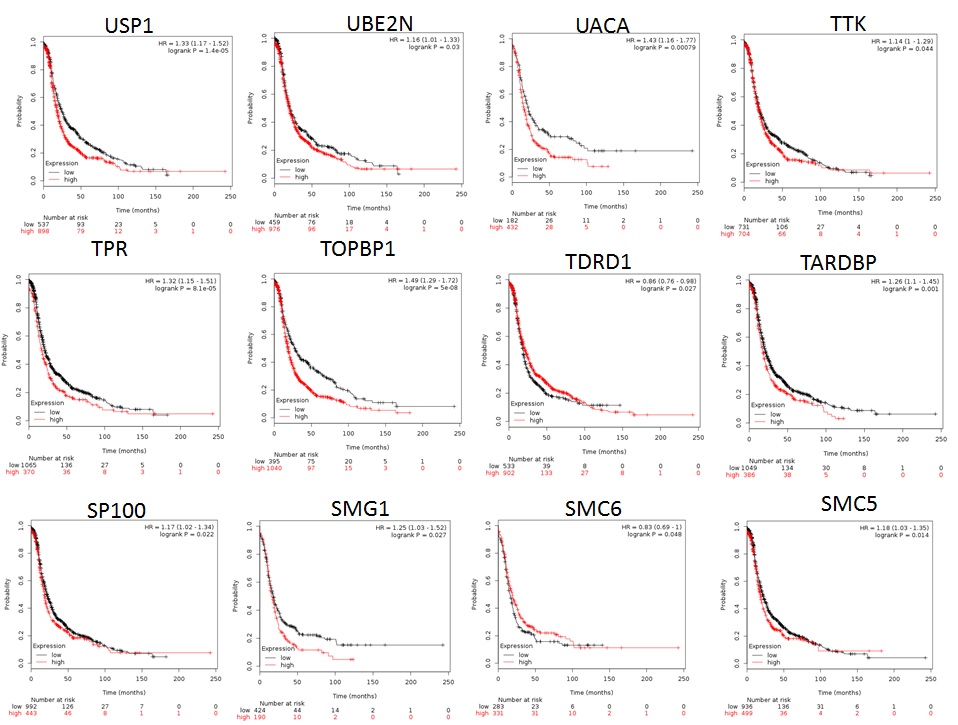


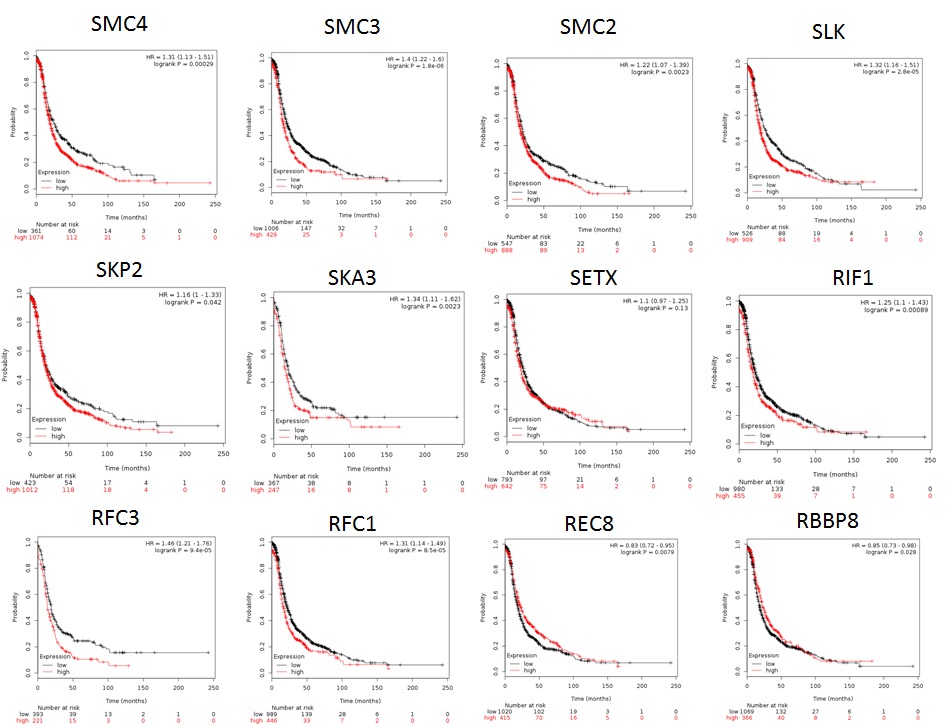


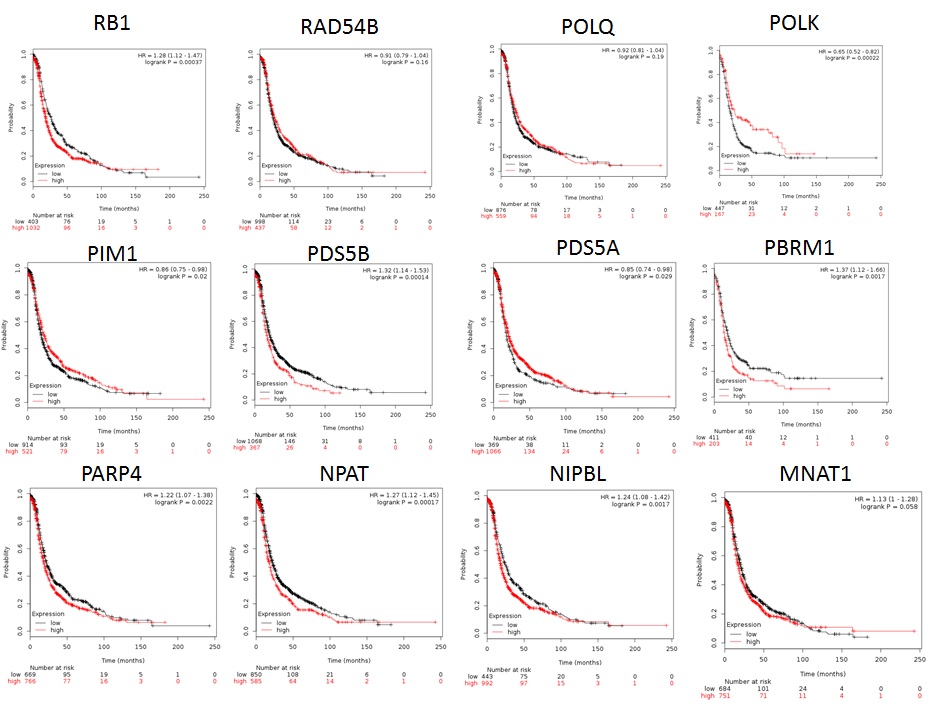


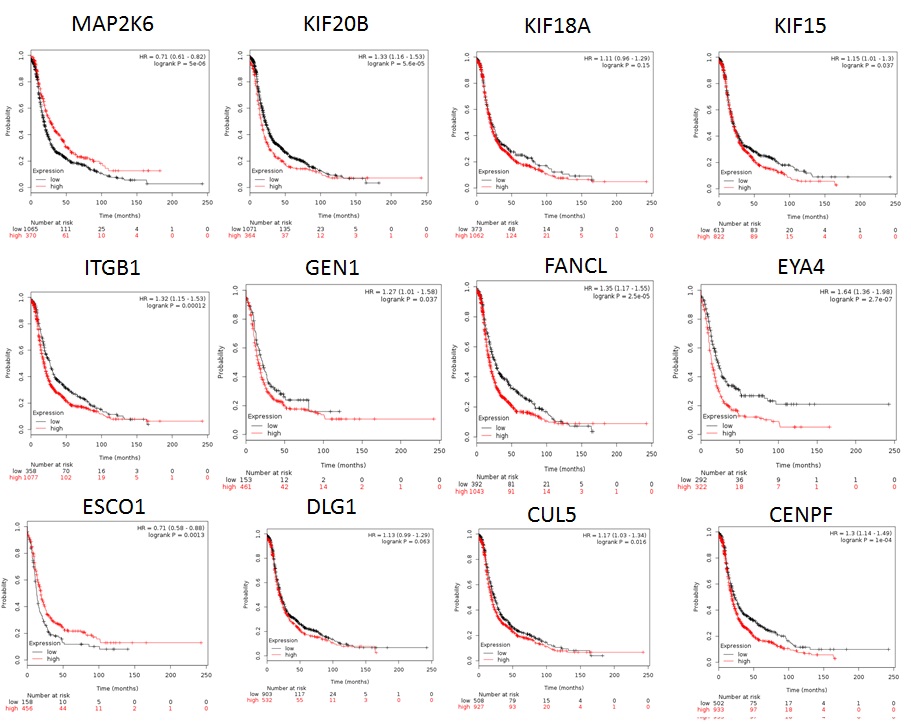


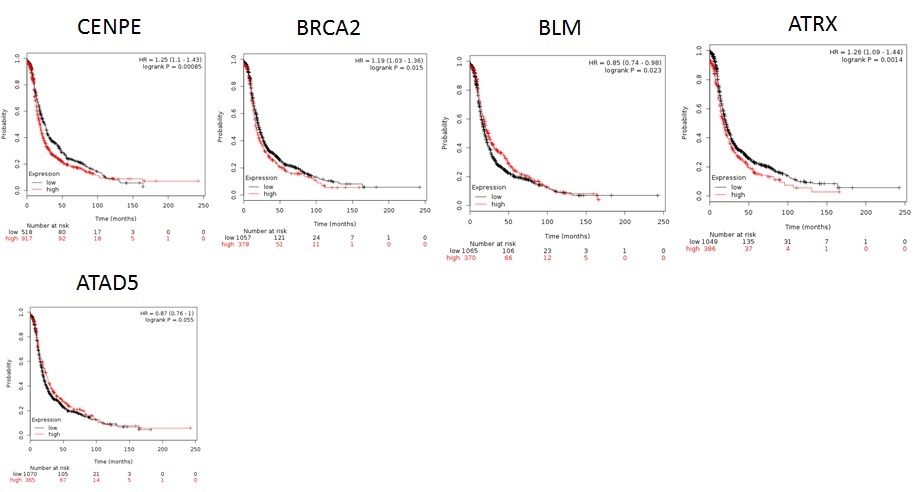


**Figure S8.1. Clinical Analysis of the Inherent Cisplatin-Resistance Gene Signature**

Each of the genes identified as an inherent cisplatin-resistance molecular signature (Table 1) was individually assessed for their potential utility as an indicator of reduced Progression Free Survival (PFS) using the online tool Kaplan-Meier Plotter. The images show the output, which indicates that 68% of the genes were statistically significant (p≤0.05). Of these, 17 had a hazard ratio of ≥1.3. These genes will be tested for their potential utility as bio-markers in future work.
